# Strategic design of wind energy and battery storage for efficient and sustainable energy systems

**DOI:** 10.1038/s41598-025-18863-5

**Published:** 2025-10-07

**Authors:** Hasan Eroğlu, Osman Kurtuluş

**Affiliations:** Faculty of Engineering and Architecture, Department of Electrical and Electronics Engineering, Recep Tayyip Erdogan University, Rize, Turkey

**Keywords:** Battery, Energy, Wind, Hybrid, Strategy, Design, Sustainable, Energy science and technology, Energy storage, Renewable energy

## Abstract

The intermittent nature of renewable energy sources, particularly wind power, necessitates advanced energy management and storage strategies to ensure grid stability and economic viability. This study investigates the techno economic benefits of integrating Battery Energy Storage Systems (BESS) into wind power plants by developing and evaluating optimized hybrid operation strategies. Using real world Data from a 70 MW wind farm, ten distinct operational strategies were simulated, incorporating approaches such as peak shaving, time shifted dispatch, and imbalance cost minimization. The battery capacity was optimized in the range of 5–70 MW. Simulation results show that battery integration reduced imbalance costs by 15–40%, while increasing total revenue by approximately 8–10%. In certain strategies, net positive total profit reached up to 60,000 USD, and the combined benefit from imbalance and revenue gains exceeded 12,000 USD under optimal conditions. These findings highlight the strategic role of BESS in enhancing system flexibility and economic return. By quantifying the relationship between control strategies and profitability, the study provides actionable insights for renewable energy operators and policy makers. It bridges the gap between theoretical hybrid models and real world deployment, supporting more resilient and efficient renewable energy integration in liberalized electricity markets.

## Introduction

### General context and importance of battery systems

Energy is an indispensable element of the modern world, and with advancing technology, the demand for energy continues to rise daily. This growing demand necessitates the development of innovative solutions not only in energy production but also in energy storage and distribution. In this context, battery energy plays a critical role in both portable and stationary energy storage systems. Batteries have the capacity to store electrical energy as chemical energy and convert it back into electrical energy when needed. These capabilities make batteries an ideal energy storage solution for integrating renewable energy sources, powering electric vehicles, portable electronic devices, and numerous other applications.

### Literature review summary

#### Manufacturing and safety

 Today, rapid advancements in battery technologies have enabled increased energy storage capacities and reduced costs. Lithium-ion batteries, one of the most widely used battery types in this field, stand out with their high energy density, long lifespan, and recyclable structures^1^. Moreover, next generation battery technologies are being developed using more environmentally friendly materials and offering higher recyclability rates. These advancements demonstrate the significant contribution of battery energy to achieving sustainability goals.

The importance of battery energy is evident not only in the efficient utilization of renewable energy systems but also in ensuring energy security^2^. Battery systems, which reduce dependency on traditional energy sources, enable the uninterrupted operation of critical infrastructure during disruptions in energy supply. Furthermore, battery technologies play a vital role in enhancing the efficiency of modern energy distribution systems, such as microgrids^3^ and smart grids^4^. These systems stabilize energy distribution by balancing fluctuations in energy demand and preventing overloads, resulting in a more reliable and stable energy network.

The widespread adoption of electric vehicles (EVs) has been a significant driver in expanding the applications of battery energy. The environmental impact and limited reserves of fossil fuels have increased the demand for clean and sustainable transportation solutions. Thanks to batteries, EVs provide a low emission and energy efficient alternative to fossil fuel powered vehicles^5^. This transformation has brought significant changes not only to the transportation sector but also to the broader energy infrastructure. Establishing the necessary charging infrastructure for EVs^6^, managing energy grids, and developing battery recycling systems are all areas that further solidify the role of battery energy in the modern world.

Studies targeting battery lifespans address both individual batteries and group battery systems. Research in^7^ explored the potential of BESS groups to enhance grid connectivity and local renewable energy consumption. A group planning strategy considering battery degradation was proposed to ensure safe and efficient operation. The strategy minimizes battery health loss and balances battery health among BESS units.

In^8^, neural network technologies were utilized to predict the remaining life of lithiumion batteries. A novel method for estimating the state of charge (SOC) was proposed, leveraging Levy flight steps to improve search efficiency while preserving the advantages of neural networks. This approach enabled more accurate SOC predictions and provided an optimized management algorithm.

The intelligent use of batteries in portable electronic devices^9^ has become an integral part of modern lifestyles. Studies show that devices such as smartphones, tablets, laptops, and wearable technologies offer extended usability thanks to battery advancements^10^. These devices are now indispensable in daily life, underscoring the need for continuous improvement and innovation in battery technologies. Factors such as increasing energy density, reducing charging times, and enhancing safety standards directly influence user experience^11^.

#### Energy sector integration

The contributions of battery energy to effective decision making processes in energy markets, operational control methods, hybrid energy systems, and many other areas are extensively documented. The current trends and advancements in this field are summarized as follows:

From an energy storage perspective, hybrid and multi energy systems are being developed to improve grid resilience and renewable energy utilization. A novel wave driven compressed air energy storage system that adapts to wave dynamics through controlled compressor operation shows high conversion efficiency and potential for offshore applications^12^. Gravity-based energy storage technologies also emerge as competitive alternatives to conventional batteries due to their simplicity, scalability, and environmental friendliness^13^. Emergency oriented storage planning under extreme weather conditions, such as hurricanes, is enhanced by robust modeling of wind power and line fault correlations using advanced optimization algorithms^14^. Furthermore, coordinated reconfiguration strategies during natural disasters support the resilience of integrated electricity and district heating systems, enabling multi-stage optimization for improved service restoration^14^. Finally, the hydrogen energy chain concept offers a new dimension for integrated energy systems, with coordinated planning across hydrogen production, storage, transport, and use, allowing for more efficient energy system design^15^.

A hybrid microgrid power system integrating wind energy, battery storage, and an Intelligent Demand Side Management System (IDSMS) was proposed in^16^. The system uses a fuzzy logic based coordinated controller aimed at improving power quality. Simulations demonstrate that the proposed control strategy effectively regulates and stabilizes frequency fluctuations and battery storage capacity in a specific region.

A stepwise learning system for decision-making in electricity markets was analyzed in^17^. Experiments on a simulated Battery Energy Storage System (BESS) reveal that the system achieves higher profitability compared to traditional methods. Particularly in volatile markets, the proposed system’s ability to quickly adapt to price changes provides a significant profit advantage over heuristic control methods. The authors highlight its potential for optimizing energy use and decision-making in energy markets.

A study in^18^ introduced a novel approach to battery storage management using a cloud based control system. This method enables more intelligent operational controls and eliminates the need for on-site operators, achieving operational gains as a result.

Research in^19^ focused on extending battery life and optimizing operational costs in hybrid energy systems. By adjusting electricity sourced from turbine output and external grids at different times, economic operation was achieved to meet user demands, while minimizing costs associated with battery degradation. The authors emphasize that future studies should focus on further optimizing and managing BESS systems, leading to smarter strategies for prolonging battery life.

Economic feasibility studies, such as in^20^, highlight how specific energy price ranges influence optimal system configurations. For example, when energy prices fall below a certain threshold, additional investments in batteries or supplementary energy restrictions may not be economically justified. Conversely, specific price ranges make battery integration economically viable for power correction purposes.

A dual group energy storage strategy was proposed in^21^ to address challenges caused by wind energy fluctuations. The first group compensates for imbalanced power and sudden loads, effectively mitigating issues. The second group employs a variable-mode adaptive frequency division method, optimizing the compensation power for both high-frequency supercapacitors and low-frequency batteries. This approach maintains the original signal’s features and reduces wind energy grid fluctuations with a two layer model.

A hybrid battery supercapacitor design was simulated in^22^, demonstrating the optimization of power distribution in energy storage units using a simple control strategy. Given the intrinsic reliance on battery systems, battery longevity remains a critical consideration in such developments.

For pulsed loads, a hybrid battery ultracapacitor energy storage system was introduced in^23^. Using a MATLAB/Simulink model, the control strategy was designed to minimize factors affecting battery lifespan.

A study proposing a two stage energy management strategy to minimize operating costs and achieve global optimization at a higher level has been presented in^24^. Additionally, studies focusing on ensuring the safe and efficient operation of systems while considering battery lifespan degradation have been conducted in^25^.

Another study takes a different approach by exploring how energy management extends to residential applications, highlighting the personal benefits of battery energy storage systems^26^.

In the study by Leonardo et al., the importance of integrating battery energy storage systems with renewable energy sources like solar energy is emphasized. Given that the efficiency of battery systems depends on the control mechanisms governing charging and discharging processes, the limitations of rule based controllers are addressed. An optimal control framework tailored to nodes with PV systems is proposed^27^.

In another study related to battery energy, a novel battery configuration technique that addresses imbalance issues in battery systems has been presented. This approach offers a fast and flexible balancing method that increases system durability and lifespan without requiring additional equipment^28^.

Studies investigating the relationship between battery capacity and lifespan have also been developed. Researchers in^29^ demonstrated that battery capacity decreases due to aging in microgrid systems, while increasing battery capacity significantly improves its health.

This study comprehensively examines interactions between buildings, transportation, and energy grids at the urban scale, highlighting opportunities for energy sharing and carbon reduction. E-mobility solutions including electric vehicles, charging stations, and hydrogen refueling infrastructure are identified as pivotal nodes in multi-sector energy systems. The integration of AI-driven decision tools and resilience indicators forms a holistic energy management framework aimed at developing sustainable and climate-resilient cities^30^.

The study compares centralized and distributed PV–battery systems, proposing a new capacity optimization method tailored for prosumer models that incorporates PV generation, building consumption, and battery aging. It evaluates total carbon intensity through lifecycle analysis and provides zero-carbon design guidelines^31^.

This research focuses on extending battery lifespan within building-centric multi energy systems through comparative analysis of thermal energy storage and depth-of-discharge (DoD)-based control strategies. It evaluates long term economic and environmental impacts of both Li-ion and lead acid batteries, using dynamic cycling aging models to link lifecycle, cost, and degradation. The study demonstrates the effectiveness of concurrent thermal electrical control strategies in slowing battery degradation and introduces novel control mechanisms^32^.

This work adopts a comprehensive approach to battery systems in multi agent energy-sharing networks, covering battery aging modeling, smart energy management, single and multi objective optimization, and energy flexibility. It includes interactive systems involving solar, wind, building, stationary and mobile batteries, and microgrids. AI-based forecasting and decision-making strategies are employed, and deterministic and stochastic optimization frameworks are implemented to handle environmental and operational uncertainties affecting battery performance^33^.

This work introduces a shared and rental energy storage planning approach for industrial parks with multiple users. It accounts for solar generation uncertainty and varied electricity demand to ensure economic and reliable system operation. Users can lease storage capacity seasonally, reducing upfront costs. Regional control and decision mechanisms limit information exchange and reduce communication costs^34^.

The transition toward sustainable energy systems has been gaining momentum across the building, transportation, and power grid sectors, closely aligned with the United Nations’ sustainable development goals. While single sector approaches have provided valuable contributions, city scale, multi sector energy systems foster stronger energy sharing and more effective carbon emission reductions. In particular, charging and hydrogen refueling stations are identified as critical nodes for urban cross-sector energy integration, underscoring the necessity of incorporating climate change impacts into the planning of such infrastructures^30^.

Within low-carbon transitions, battery systems act as an essential bridge between renewable energy generation and demand. However, improper sizing methods in both centralized and distributed systems often lead to performance misestimations and resource inefficiencies. To address this challenge, renewable-storage sizing approaches are becoming critical for ensuring long-term sustainability. Moreover, artificial intelligence assisted storage optimization, based on big data modeling and predictive algorithms, is expected to play an increasingly important role in efficient system design^35^.

Hybrid hydrogen battery systems have also emerged as a promising solution for stabilizing the intermittent nature of renewables, mitigating grid fluctuations, and enhancing system resilience. By compensating for the idling losses of hydrogen systems, battery integration improves both technical reliability and economic feasibility. Recent studies further introduce innovative indicators to evaluate the techno economic performance of such hybrid systems, highlighting the dependence of feasibility on the balance between hydrogen and battery capacities^36^.

At the district level, building vehicle interactive energy networks are increasingly recognized as an effective means of enhancing clean energy penetration and overall system performance. Nevertheless, the higher frequency of charging and discharging cycles accelerates battery aging. To overcome this issue, dynamic self learning grid responsive strategies have been proposed, improving battery lifetime and enabling more balanced trade offs between costs and energy flexibility through multi objective optimization methods^37^.

Finally, peer to peer (P2P) energy trading models are gaining attention in net zero energy communities. Integrated systems combining solar, wind, and hydrogen storage technologies within university campuses, office complexes, and high rise residential districts enhance local energy self consumption while reducing grid dependence. Individualized pricing mechanisms and time of use management strategies further improve renewable energy utilization, reduce costs, and significantly cut carbon emissions, thereby offering practical pathways for large scale urban applications^38^.

#### Wind and battery energy sector integration

Recent advances in wind power generation focus on maximizing energy extraction, enhancing control strategies, and improving forecast reliability. For offshore wind turbines, a two-degree-of-freedom loop shaping robust control design with individual pitch control has proven effective in improving power output and system stability while mitigating structural loads^39^. Additionally, the availability of high-quality wind power data plays a vital role in production planning and prediction. Hybrid data reconstruction methods combining clustering-based anomaly detection, bidirectional gated recurrent units, and ensemble learning help eliminate anomalies and boost the accuracy of forecasting models^40^. Moreover, the trustworthiness and interpretability of machine learning models for wind power forecasting are increasingly emphasized. Explainable techniques, such as local interpretable model agnostic explanations, enable risk assessment and improve model transparency across seasons and time horizons^41^. On a structural safety level, novel quantile formulas for estimating extreme wind pressures allow for more accurate infrastructure design in wind power systems, incorporating mean wind speed variations and recurrence intervals^42^.

This study develops a mathematical model to optimally size and assess an off grid hybrid power system based solely on variable renewable energy sources and hybrid storage. Using Ometepe Island as a case study, the model evaluates how capital cost variations and power supply reliability affect energy cost. Results highlight that utilizing a volcanic crater lake as a pumped hydro reservoir significantly improves system feasibility, with cost effective and reliable energy outcomes^43^.

Battery lifecycle strategies for emission reduction such as V2X (with multi-directional Vehicle to Everything) interactions and cascade use have been proposed in literature, showing environmental and economic benefits in renewable integrated systems. In this study, these approaches are extended to hybrid wind battery systems: capacity optimization under varying operational strategies reveals their profit potential, offering practical contributions to the field^44^.

This study investigates control and energy management strategies for hybrid renewable energy systems combining wind and solar power with battery storage. By employing Maximum Power Point Tracking (MPPT) algorithms alongside advanced control techniques, the system’s overall efficiency is enhanced. Simulation results validate system performance and component design under varying operational conditions, demonstrating the practical applicability of the proposed approach^45^.

This study presents a comprehensive literature review on control strategies used in battery energy storage systems (BESS) to smooth out wind power fluctuations. While wind energy has become increasingly prominent in the global energy mix, its intermittent nature affects power quality and grid stability. BESS is commonly employed to mitigate these fluctuations, but large storage capacity demands drive up costs. To address this, control systems have been proposed to optimize BESS use and reduce its size. The review finds that most existing studies utilize PI, fuzzy logic, and model predictive control (MPC) techniques, with relatively limited exploration of deep learning based approaches an emerging area with significant potential in wind energy applications. As the scope of this review suggests, there is a clear development trend in the literature in this direction, and research efforts continue to expand in this area^46^.

In line with this trend, another study explores the integration of battery storage systems with wind energy by developing a power management control (PMC) strategy combined with a two-level maximum power point tracking (MPPT) algorithm. The proposed control framework optimizes energy extraction while reducing stress on the battery storage units. Through Matlab/Simulink based simulations using experimentally identified wind turbine parameters, the study demonstrates that the hybridization of torque and speed based MPPT algorithms with PMC significantly improves overall system efficiency, response time, and battery state of charge performance. The findings highlight the system’s potential for more effective and sustainable wind energy utilization^47^.

### Research gap & motivation

The hybridization of wind energy and battery storage systems represents a pivotal advancement in the renewable energy sector, promising enhanced supply stability and improved grid reliability. Integrating these systems can effectively address the imbalance between variable generation and demand by enabling multi dimensional operational strategies ranging from power smoothing and time shifted dispatch to real time load balancing and battery capacity optimization. However, despite these developments, the existing body of literature remains predominantly focused on theoretical modeling or limited to technical benefits, often overlooking comprehensive economic analyses.

In particular, there is a notable lack of strategy-based evaluations grounded in real-world data examining the techno economic performance of Battery Energy Storage Systems (BESS) integrated into large scale wind farms. Comparative studies that analyze different control strategies such as peak shaving, imbalance cost mitigation, and time shifted operation, while also considering battery capacity optimization, are scarce. This gap hinders decision-makers from accessing practical, evidence based insights necessary for guiding investments and operational planning in renewable energy systems.

The need for such studies is further highlighted in Table 1, which provides a structured summary of the existing literature. The table outlines the scope, methodologies, and limitations of prior studies, thereby underscoring the importance of comprehensive, data driven evaluations that incorporate both technical and economic dimensions of wind BESS hybrid systems.

**Table 1:** Comparative overview of battery storage strategies in the energy sector.

Refs.	Study Focus	Methodology	Key Strategy	Limitations/Gaps
[12]	Wave-driven CAES	Adaptive compressor control	High offshore efficiency	Limited scalability
[13]	Gravity energy storage	Mechanical potential energy	Low environmental impact	Low energy density
[14]	Hurricane-resilient storage	Robust optimization	Grid resilience	Conservative design
[15]	Multi-stage grid reconfiguration	Integrated power-heating networks	Service restoration	High complexity
[16]	Hydrogen energy chain	Multi-system collaborative planning	Holistic integration	High infrastructure costs
[17]	Wind-battery-IDSMS microgrid	Fuzzy logic control	Frequency stabilization	Slow dynamic response
[18]	BESS for electricity markets	Reinforcement learning (RL)	Price volatility adaptation	Computationally intensive
[19]	Cloud-based BESS control	Remote monitoring	Operator-free operation	Cybersecurity risks
[20]	Battery lifetime extension	Degradation-cost optimization	Economic operation	Ignores real-time pricing
[21]	Wind turbine power smoothing	Adaptive pitch + batteries	Reduced mechanical wear	Battery cycling stress
[22]	Dual-group storage (battery + SC)	Frequency division control	Wind fluctuation mitigation	Complex control
[23]	Hybrid battery-SC design	Multistage energy management	Power sharing	Limited scalability
[24]	Battery-ultracapacitor for pulsed loads	MATLAB/Simulink modeling	Lifespan preservation	High ultracapacitor costs
[25]	Two-stage energy management	Global cost optimization	Operating cost reduction	High computational load
[26]	Battery lifespan degradation	Aging-aware scheduling	Safe operation	Simplified aging models
[27]	Residential BESS	IoT-enabled energy monitoring	User-centric benefits	Small-scale focus
[28]	PV-battery optimal control	Rule-based vs. optimal control	Efficiency improvement	Limited to PV systems
[29]	Modular battery balancing	Fast imbalance correction	System lifespan extension	Requires modular architecture
[30]	Battery capacity-lifespan link	Aging analysis in microgrids	Health-aware operation	Empirical validation needed
[31]	Urban energy-carbon nexus	Multi-sector AI-driven framework	Energy-sharing opportunities	Macro-scale focus
[32]	PV-battery lifecycle carbon	Capacity optimization + aging	Zero-carbon design	Limited to PV
[33]	Multi-energy storage control	Thermal-electrical synergy	Battery degradation reduction	Complex implementation
[34]	Multi-agent energy sharing	Stochastic optimization	Flexibility under uncertainty	High communication overhead
[35]	Battery manufacturing and sustainability	Life-cycle assessment	Reducing carbon footprint in battery production	Limited large-scale application data
[36]	Battery safety and thermal management	Experimental and modeling approaches	Enhancing safety through advanced cooling	High costs of implementation
[37]	Integration of batteries in the energy sector	System-level simulations	Improving renewable integration	Grid complexity challenges
[38]	Wind and battery storage integration	Hybrid system analysis	Enhancing stability of wind power	Uncertainty in long-term performance
[39]	Market and policy aspects of battery adoption	Policy review and economic analysis	Encouraging large-scale adoption	Regulatory and policy gaps
[40]	Shared rental storage	Robust optimization	Cost reduction for multi-user parks	Seasonal variability
[41]	Offshore wind turbine control	2-DOF robust control	Power output + load mitigation	Ignores battery degradation
[42]	Wind data reconstruction	GRU + ensemble learning	Forecasting accuracy	Computationally heavy
[43]	Explainable wind forecasting	LIME interpretability	Model transparency	Short-term only
[44]	Extreme wind pressure	Quantile formulas	Infrastructure safety	Static wind models
[45]	Off-grid hybrid system	Pumped hydro + battery sizing	Cost-reliability tradeoff	Site-specific
[46]	Wind-battery lifecycle	V2X + cascade use	Emission reduction	Grid congestion ignored
[47]	Wind-PV-battery control	MPPT + hybrid algorithms	Efficiency optimization	Battery stress during peaks
[48]	BESS for wind smoothing	Review of PI/Fuzzy/MPC methods	Reduced BESS size	Limited deep learning
[49]	Wind-battery PMC	Torque/speed MPPT + SOC management	Fast response	Inflexible energy sharing

Recent advancements in energy storage and wind battery hybrids demonstrate significant progress in grid resilience, operational efficiency, and renewable integration. Studies like^12^ (wave-driven CAES) and^13^ (gravity storage) explore alternative storage technologies, while^17,22^ optimize battery-centric hybrid systems through fuzzy logic, reinforcement learning, and dual-group storage strategies. However, critical gaps persist, including battery degradation under dynamic loads^21,24^, inflexible energy-sharing modes^48^, and limited degradation aware control in wind-battery systems^40^. Wind energy integration further faces challenges in forecasting accuracy^41^, explainability^42^, and peak-load battery stress^46^.

### Our contribution

This study Makes significant contributions to both the academic Literature and the practical deployment of hybrid renewable energy systems. Building upon real operational Data from a 70MW wind farm, a comprehensive simulation framework is developed to evaluate ten distinct Battery Energy Storage System (BESS) control strategies. These include approaches such as peak shaving, time-shifted dispatch, and imbalance cost mitigation. Each strategy is assessed not only from a technical perspective such as reducing power fluctuations^48^, ensuring continuous and uninterrupted load supply^49^, and enabling proper utilization of battery lifespan^50^ but also through a detailed economic lens that includes operational costs and revenue streams.

By optimizing battery capacity in the range of 5–70 MW, the study identifies the most economically viable configurations, with simulation results indicating a 15–40% reduction in imbalance costs and an 8–10% increase in total revenues. Under favorable conditions, net profits reach up to 60,000 USD. These results are particularly relevant for investors and system operators seeking financially sound and technically robust solutions. Furthermore, this study offers strategic insights and practical recommendations for the optimal design and management of wind-BESS hybrid systems, effectively bridging the gap between theoretical models and real world implementation. It contributes to the development of more sustainable, cost effective, and reliable renewable energy integration by demonstrating how optimized wind-battery hybrid systems can reduce curtailment, enhance grid flexibility, and maximize renewable energy utilization.

## Hybrid wind storage systems

### Structure of the hybrid wind storage system

**Fig. 1:**
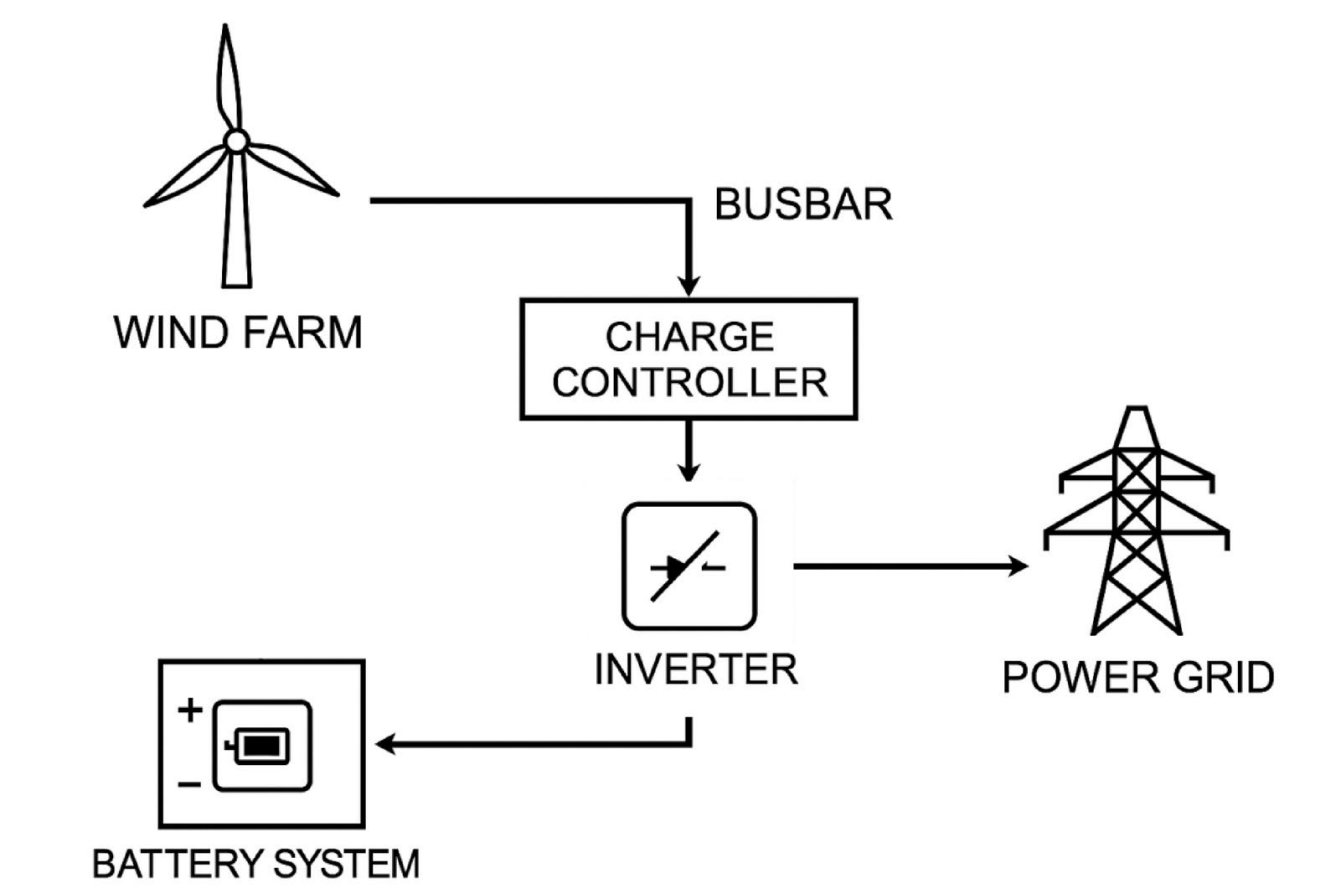
Schematic of a battery supported hybrid wind power generation facility^53^.

Energy storage systems are an essential cornerstone for smart energy and zero emission goals in the developing world^51^. Wind energy, with its existing potential, has a structure that can be developed alongside battery systems^52^. Hybrid wind storage systems are complex structures developed to balance fluctuations in wind energy production and improve energy efficiency. These systems typically include a wind power plant and a battery storage system. While the wind power plant generates electricity from wind turbines, the battery system simultaneously stores this electricity and supplies it back when needed. In this way, energy fluctuations caused by the variable nature of wind are mitigated, and system stability is maintained. An example model is shown in Fig. 1.

The battery system not only balances the fluctuations in wind energy production but also responds to changes in energy demand over time. By storing energy from the wind farm, the battery can supply additional power during peak demand periods or store surplus energy for later use when overproduction occurs. This approach helps smooth out energy consumption fluctuations and increases energy efficiency. Future studies will focus on exploring the performance of different battery technologies to balance renewable energy variability and enhance the resilience of power systems^54^.

Hybrid wind storage systems are often integrated with local electricity grids^55^. Through this integration, excess energy from wind farms can be fed into the grid, or energy from the grid can be used to meet demand. This enhances grid stability and promotes the use of renewable energy sources.

The sizing of battery supported systems is equally crucial. It significantly affects overall profitability and is vital for battery longevity^56^. Calculations performed for stable operations demonstrate a positive contribution to future outcomes.

### Modeling of energy storage characteristics

Energy storage is an effective method for shifting energy usage over time, storing surplus energy, and sending it back to the system when needed. Energy storage relies on several scientific principles^57^. Additionally, the battery systems used in energy plants must be long-lasting^58^. Another critical factor is cost. The fact that batteries will eventually reach the end of their service life translates to costs^59^. A review of the literature shows that these sensitivities have been prioritized, and systems have been designed using various learning methods^17^.

Considering all these factors, this article proposes a hybrid structure called Battery A, designed for energy storage in wind farms. Hybrid energy storage is employed to optimize wind power output and ensure efficient energy utilization.

Studies have discussed the minimum cost analysis (MinCA) required for a battery facility^21^. Building on this, given the critical importance of minimizing costs, MinCA must be factored into the proposed analysis. In examining this structure, it becomes clear that investments should not solely focus on equipment costs; the land cost ($\:{C}_{LC}$) of the proposed site must also be considered. Many strategically advantageous locations remain undeveloped due to high land costs. Consequently, MinCA has been newly formulated in this study as shown in Eq. (1).

1 $$\:minCA{\prime\:}=\frac{{r(1+r)}^{yA}}{{(1+r)}^{yA}-1}\cdot\:(Cbp\text{}\cdot\:PA+Cbe\text{}\cdot\:EA+Crb\text{}\cdot\:EA)+{C}_{LC}$$

$\:r$ Annual interest rate.

$\:yA$ Service life of Battery A.

$\:Cbp$ Unit power cost of the battery (EU/kWh).

$\:Cbe$ Unit capacity cost of the battery (EU/kWh).

$\:Crb$ Maintenance cost of the battery (EU/kWh).

$\:EA$ Capacity of Battery A.

$\:PA$ Power of Battery A.

$\:{C}_{LC}$ Land cost of the investment (EU).

The expression for power in a wind farm can be expressed Eq. (2).

2 $$\:P\left(t\right)=\:{P}_{1}\left(t\right)+{P}_{2}\left(t\right)+{P}_{3}\left(t\right)+{P}_{4}\left(t\right)+{P}_{5}\left(t\right)+.......{P}_{n}\left(t\right)+{P}_{A}\left(t\right)$$

The expression from 1 to n represents the instantaneous power of each turbine in a wind power plant. $\:{P}_{A}\left(t\right)$ represents the instantaneous power sent by Battery A to the system or drawn from the system. Therefore, Eq. (2) can be rearranged and presented more clearly as Eq. (3).

3 $$\:P\left(t\right)=\sum\:_{x=1}^{n}{P}_{x}\left(t\right)+{P}_{A}\left(t\right)$$

The financial current revenue expression for the **E** production at time **t** in a production plant is:

4 $$\:\beta\:\left(t\right)=E\left(t\right).C\left(t\right)$$

The expression $\:C\left(t\right)$ represents the electricity market price at time **t**.

The imbalance quantity is a critical metric that must be controlled for production facilities. It reflects a system’s stability, continuity, and reliability. Production facilities are obligated to operate according to a predefined plan. Deviations from this plan either overproduction or underproduction—result in penalties. Positive imbalances occur when production exceeds the planned amount, while negative imbalances arise when production falls short of the plan.

5 $$\:\gamma\:=\left\{\begin{array}{c}x.y.\left(\text{0,97}\right)-x.z,\:\:\:\:\:\:\:\:\:\:\:x>0\\\:x.y.\left(\text{1,03}\right)-x.z,\:\:\:\:\:\:\:\:\:\:\:\:\:\:x<0\end{array}\right.$$

X is the imbalance amount (MWh),

Y is the minimum value between the hourly MCP and SMP prices,

Z is the MCP price.

6 $$\:\gamma\:=\left\{\begin{array}{c}\left(E-{E}^{{\prime\:}}\right)\times\:\left(\text{0,97}\right)\times\:min\left(Z:W\right)-\left(E-{E}^{{\prime\:}}\right)\times\:Z,\:\:\:\:\:\:\:\:(E-E{\prime\:})>0\\\:\left(\text{E}-{\text{E}}^{{\prime\:}}\right)\times\:\left(\text{1,03}\right)\times\:max\left(Z:W\right)-\left(E-{E}^{{\prime\:}}\right)\times\:Z,\:\:\:\:\:\:\:\:(E-E{\prime\:})<0\end{array}\right.$$

In light of these considerations, a wind power plant should remain net positive over a given time period **t**. The length of **t** may vary depending on the nature of the investment and investor preferences, but the fundamental expectation is that an investment should meet Eq. (7) condition.

7 $$\:\sum\:_{t=0}^{t}\left(\beta\:\right(t)-minCA-\gamma\:)>0$$

Based on this principle, investments that consistently fall below the net-zero point raise concerns about their future viability and are less likely to attract further investment.

Beyond the initial costs of an investment, determining the operational framework is equally critical. For an investment to be sustainable in the long term and to achieve the expected returns, it is essential not only to manage upfront expenses but also to operate the facility efficiently. This is particularly important for innovative energy projects such as hybrid battery wind power plants. Careful planning is required for the facility’s operation, including when it will be active or on standby.

This planning process must take into account several factors, such as fluctuations in energy demand, weather conditions, and optimal utilization of battery capacity. A poorly devised operational strategy can lead to energy losses, unnecessary cost increases, and reduced returns on investment.

To mitigate these risks, detailed analyses must be conducted from the feasibility stage, and the most suitable operational strategy should be identified. Within this study, five different strategies have been developed. The details of these strategies are provided in the following sections.

## Development of strategies for battery A

Battery facilities are critical infrastructures designed to store electrical energy and deliver it when needed. For these facilities to operate efficiently, charging and discharging strategies are crucial. Moreover, intelligent battery systems have been developed to manage their complex structures effectively^60^. Charging strategies aim to enhance the battery’s energy storage capacity, while discharging strategies ensure the stored energy can be supplied to the system as needed. Machine learning-based systems have also been developed to manage these processes accurately^61^.

**Fig. 2:**
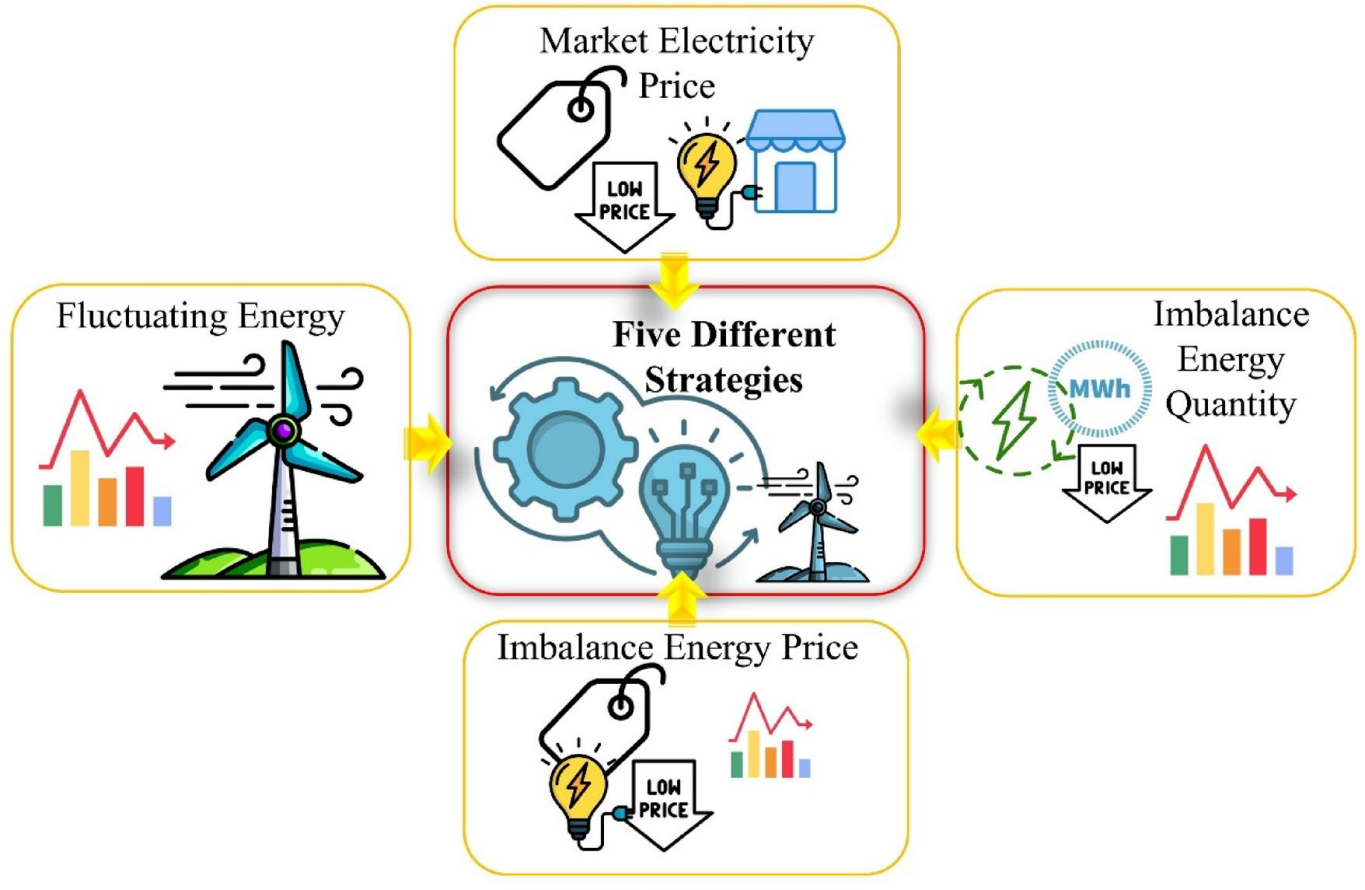
Battery-supported energy market and operating strategies.

In this study, multi-stage optimal charging and discharging strategies for a battery facility were developed. Properly designing and implementing these strategies can enhance the performance of energy storage systems, optimize energy efficiency, and ensure the stability of electrical grids. The framework incorporating the various strategies established for this study is illustrated in Fig. 2.

**Fig. 3:**
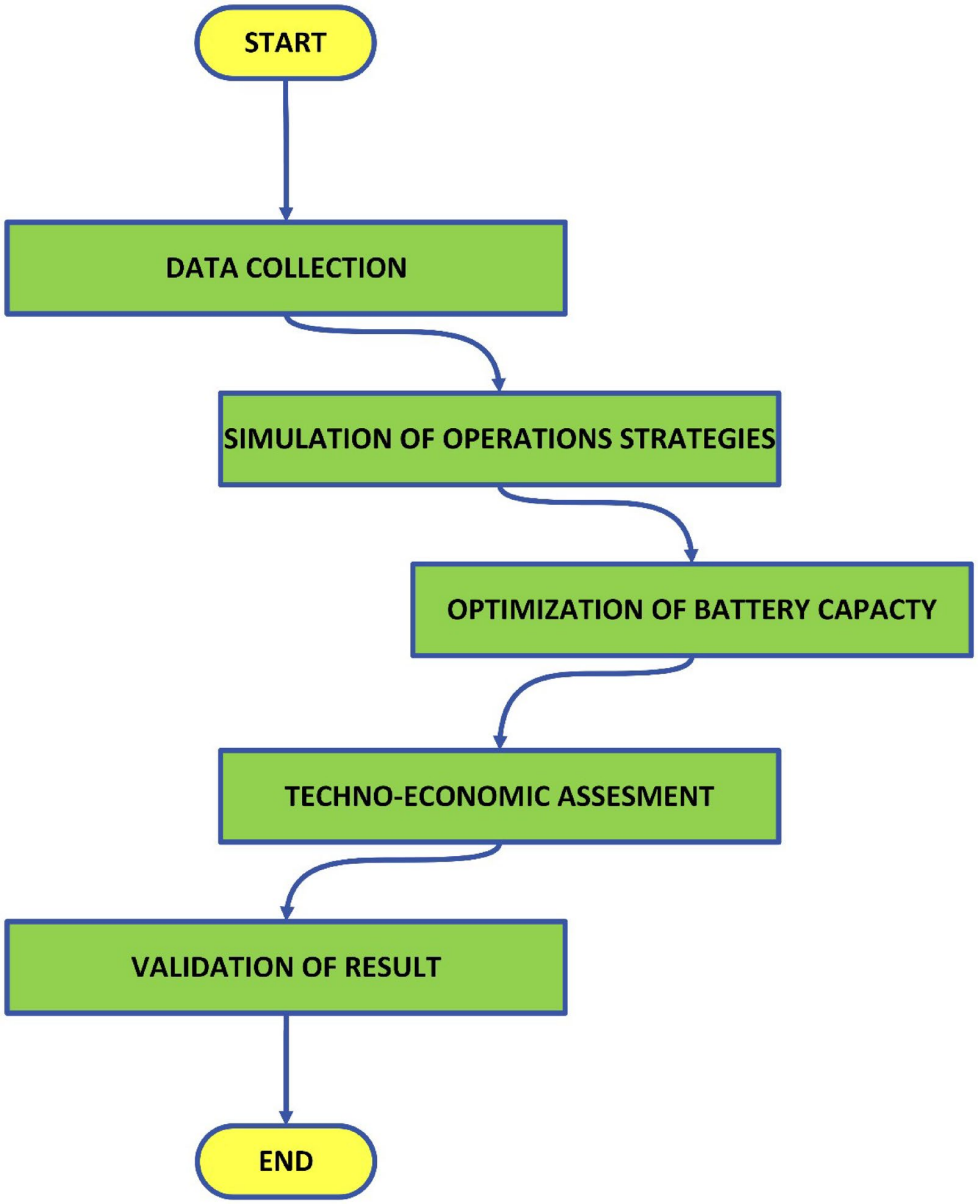
Schematic representation of the study methodology.

The operational workflow designed for this study is illustrated in Fig. 3. It begins with data collection, which forms the foundation for the subsequent steps. This is followed by the simulation of operational strategies, allowing the assessment of various battery usage scenarios. Next, the optimization of battery capacity step aims to determine the most efficient storage configuration. A techno economic assessment is then conducted to evaluate the feasibility of the strategies from both technical and financial perspectives. Finally, the process concludes with the validation of results, ensuring the reliability and applicability of the developed strategies in real world battery energy storage systems.

### Proposed operational strategy for stabilizing fluctuating energy delivered to the grid

The smoothing method for wind energy output ensures that the energy delivered to the grid is maintained at a stable level. This approach can also be refined depending on the transmission distance^62^. Strategies for stabilizing fluctuating energy delivered to the grid involve techniques to regulate the variable electricity production from renewable energy sources and optimize its integration into the grid.

**Fig. 4:**
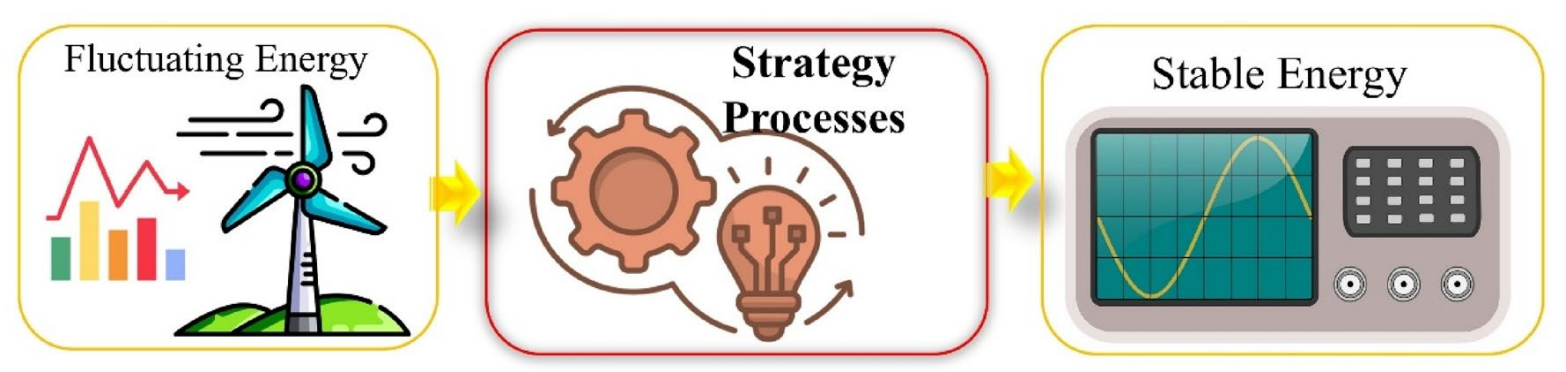
Schematic representation of the proposed strategy.

These strategies include the use of accumulator batteries, the integration of energy storage systems, smart grid management, and power electronics-based correction techniques. These methods improve the efficiency of renewable energy sources integrated into the grid, making energy flow more stable and enhancing grid reliability. The framework for the strategy defined in this study is presented in Fig. 4.

The battery charging strategy, when expressed as a more general mathematical equation, is as Eq. (8):

8 $$\:E\left(t\right)=\text{m}\text{i}\text{n}\left(\text{m}\text{a}\text{x}\right(E(t-\varDelta\:t)+{\varDelta\:E}_{ekle}\text{},{E}_{min}\text{}),{E}_{max}\text{})$$

Where:

- $\:E\left(t\right)$ represents the energy level in the battery at time t (MWh).
- $\:\varDelta\:t$ represents the time step (t).
- $\:{\varDelta\:E}_{ekle}$​ add denotes the amount of energy to be added or removed from the battery. This amount can be positive or negative depending on the charging or discharging strategy (MWh).
- $\:{E}_{min}$​ represents the minimum capacity of the battery. The battery cannot fall below this value (MWh).
- $\:{E}_{max}$​ represents the maximum capacity of the battery. The battery cannot exceed this value (MWh).

**Fig. 5:**
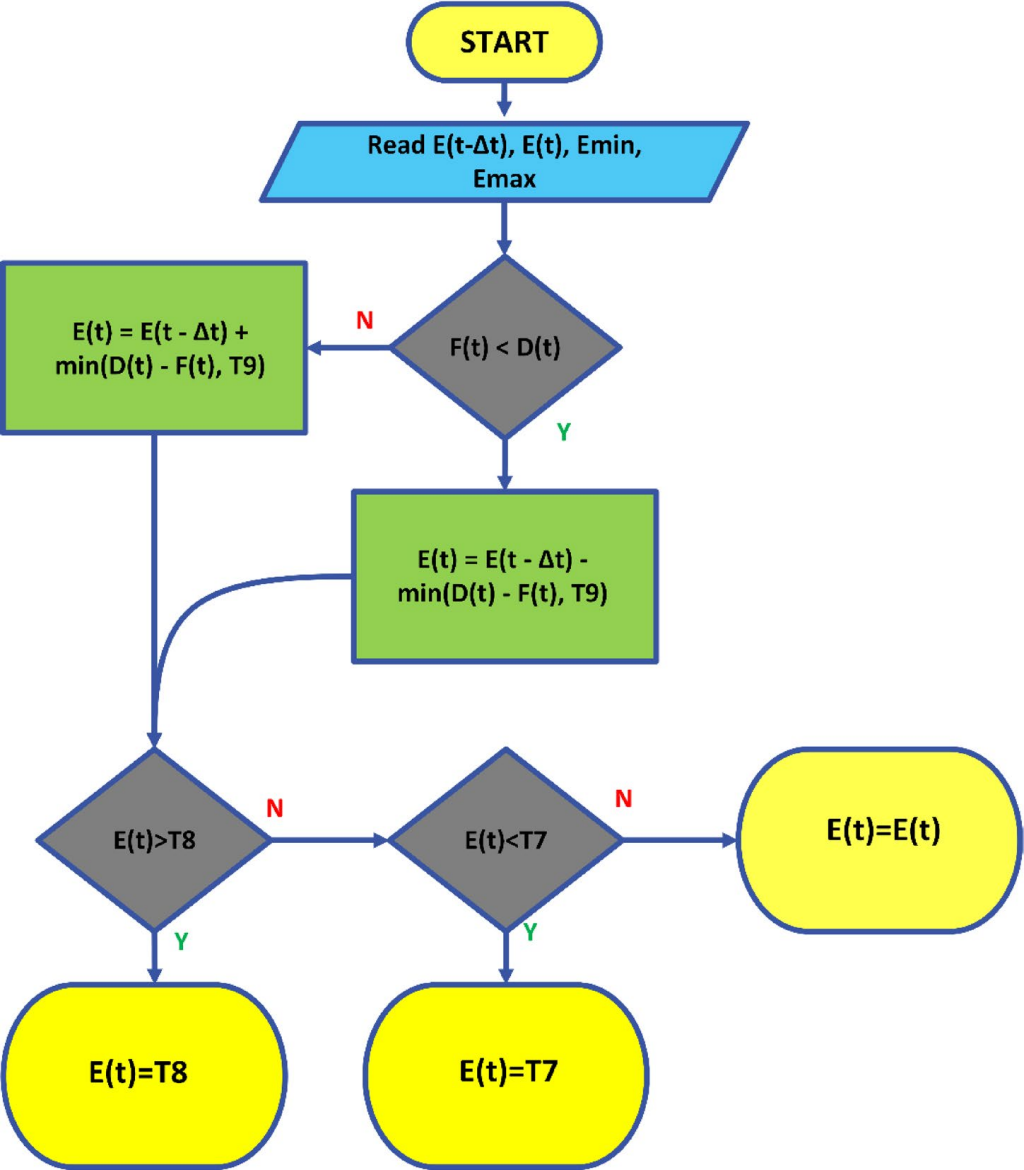
The flowchart of the proposed strategy.

In the governing equation, the new energy level of the battery $\:E\left(t\right)$ is calculated based on the previous time step’s energy level $\:E(t-\varDelta\:t)$ and the energy amount $\:{\varDelta\:E}_{ekle}$​ added or subtracted. The resulting value is constrained within the range of $\:{E}_{min}$​ and $\:{E}_{max}$​. This ensures the battery remains within a specific range, keeping the charging/discharging operations under control. The flow diagram related to this strategy is depicted in Fig. 5.

### Proposed operational strategy based on market electricity prices

**Fig. 6:**
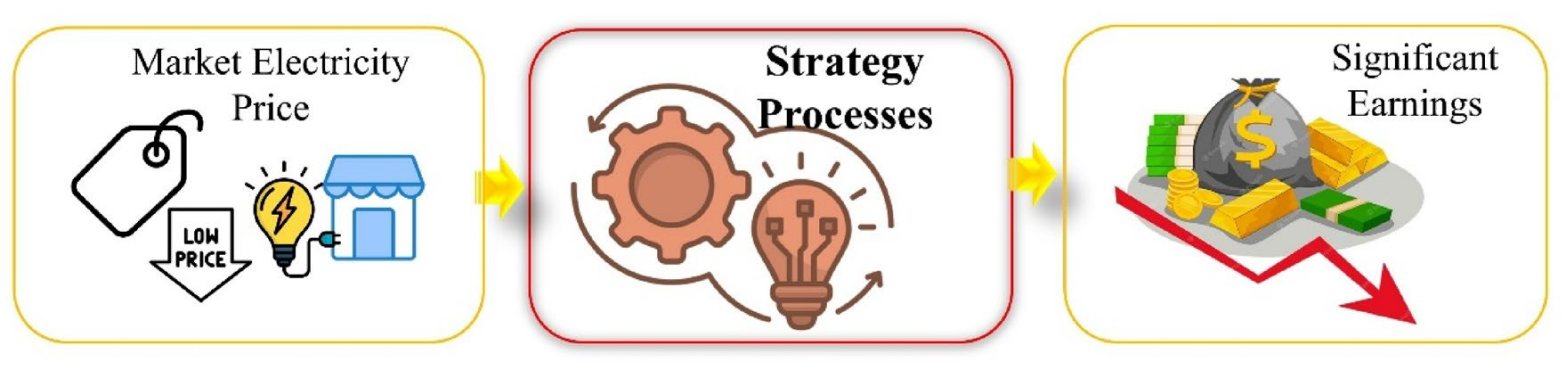
Schematic representation of the proposed strategy.

In the previous section, a power smoothing operation at the plant output on a power basis was performed, entirely independent of price. However, in some cases, price becomes a more significant factor. For example, when there is an energy demand in the electrical grid, storing excess energy in the battery instead of delivering it to the system could negatively impact operational costs. Therefore, under this section, a storage strategy based on price has been developed and is illustrated in Fig. 6.

9 $$\:E\left(t\right)=min\left\{max\left\{\left\{\begin{array}{c}E(t-\varDelta\:t)+min({\text{T}}_{9},F(t\left)\right),\:\:\:\:\:\:\:H\left(t\right)<{\text{T}}_{14}\\\:E(t-\varDelta\:t)-min({\text{T}}_{9},E(t\left)\right),\:\:\:\:\:\:\:H\left(t\right)>{\text{T}}_{15}\\\:E(t-\varDelta\:t),\:\:\:\:\:\:\:\:\:\:\:\:\:\:\:\:\:\:\:\:\:\:\:\:\:\:\:\:\:\:\:\:\:\:\:\:\:\:\:otherwise\end{array}\right.\right\},\text{E}\text{m}\text{i}\text{n}\right\},\text{E}\text{m}\text{a}\text{x}$$

- $\:\text{H}\left(\text{t}\right)$ Market electricity price at time t (USD).
- $\:\text{F}\left(\text{t}\right)$ Amount of energy to be added to the battery at time ttt (USD).
- $\:{\text{T}}_{7}$ The lower energy limit of the battery: If this limit is breached, the battery reaches a critical level and requires immediate charging or intervention (MWh).
- $\:{\text{T}}_{8}$​ The maximum energy limit of the battery: If this limit is exceeded, the battery is overcharged and may sustain damage (MWh).
- $\:{\text{T}}_{9}$ The maximum amount of energy that can be added to or removed from the battery (MWh).
- $\:{\text{T}}_{14}$​ and $\:{\text{T}}_{15}$​ Price thresholds. If $\:\text{H}\left(\text{t}\right)<{\text{T}}_{14}$​ charging occurs. H $\:\text{H}\left(\text{t}\right)>{\text{T}}_{15}$​ discharging occurs (USD/hr).

**Fig. 7:**
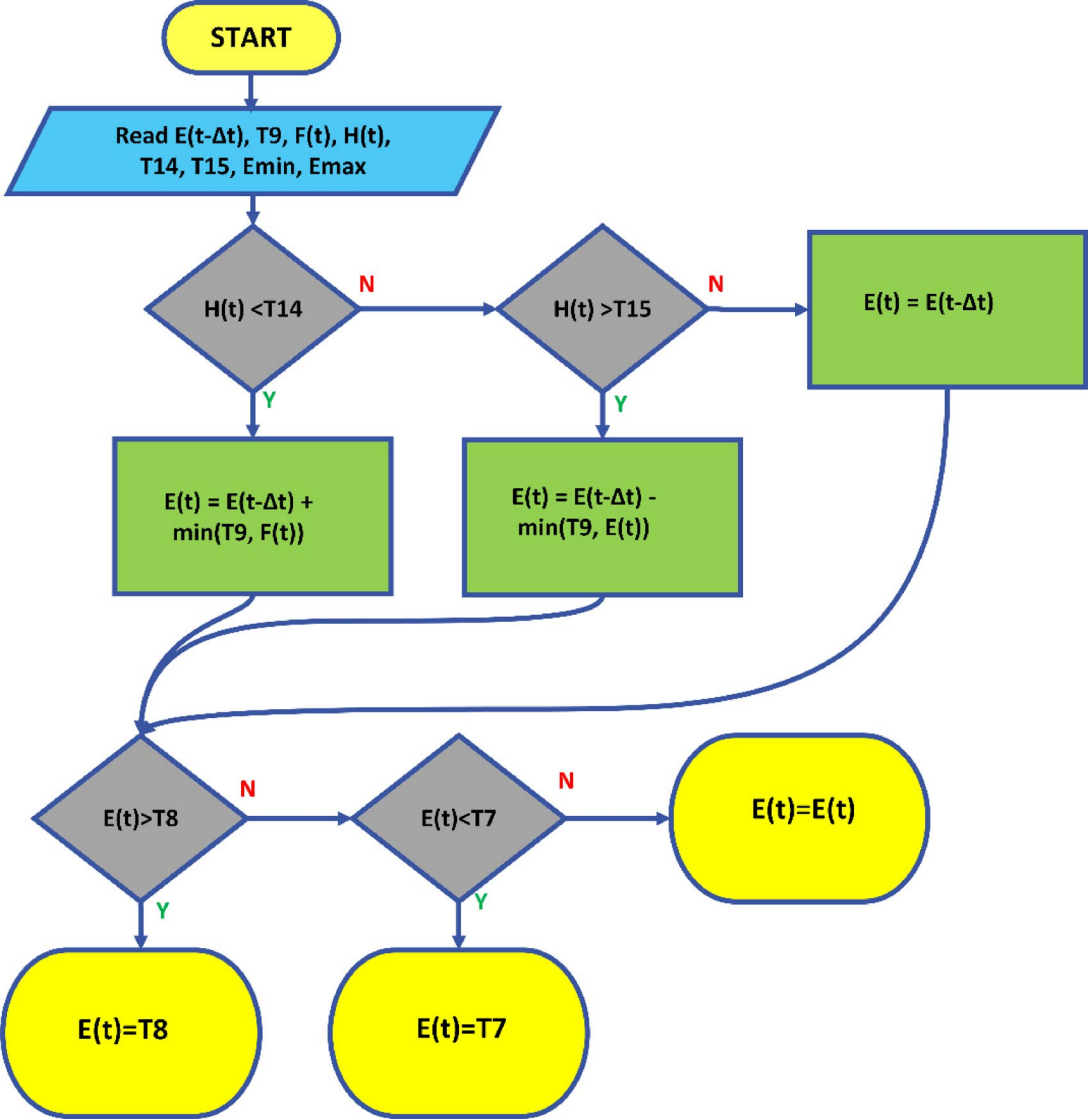
The flowchart of the proposed strategy.

If $\:\text{H}\left(\text{t}\right)<{\text{T}}_{14}$​ the battery’s energy level should be increased. In this case, the smaller value between the current energy level $\:\text{E}(\text{t}-{\Delta\:}\text{t})$ and the minimum energy amount to be added is chosen. If the minimum energy amount to be added exceeds the $\:{\text{T}}_{9}$​ limit, only $\:{\text{T}}_{9}$​ worth of energy is added.

If $\:\text{H}\left(\text{t}\right)>{\text{T}}_{15}$​ energy needs to be discharged from the battery. In this scenario, the smaller value between the current energy level $\:\text{E}(\text{t}-{\Delta\:}\text{t})$ nd the minimum energy amount to be removed is selected. If the minimum energy amount to be removed exceeds the $\:{\text{T}}_{9}$​ limit, only $\:{\text{T}}_{9}$​ worth of energy is discharged.

If none of these conditions are met, the energy level of the battery remains unchanged, and the current energy level $\:\text{E}(\text{t}-{\Delta\:}\text{t})$ is maintained.

The flow diagram of the proposed strategy is presented in Fig. 7.

### Proposed operational strategy based on simultaneous evaluation of imbalance MWh and market price

**Fig. 8:**
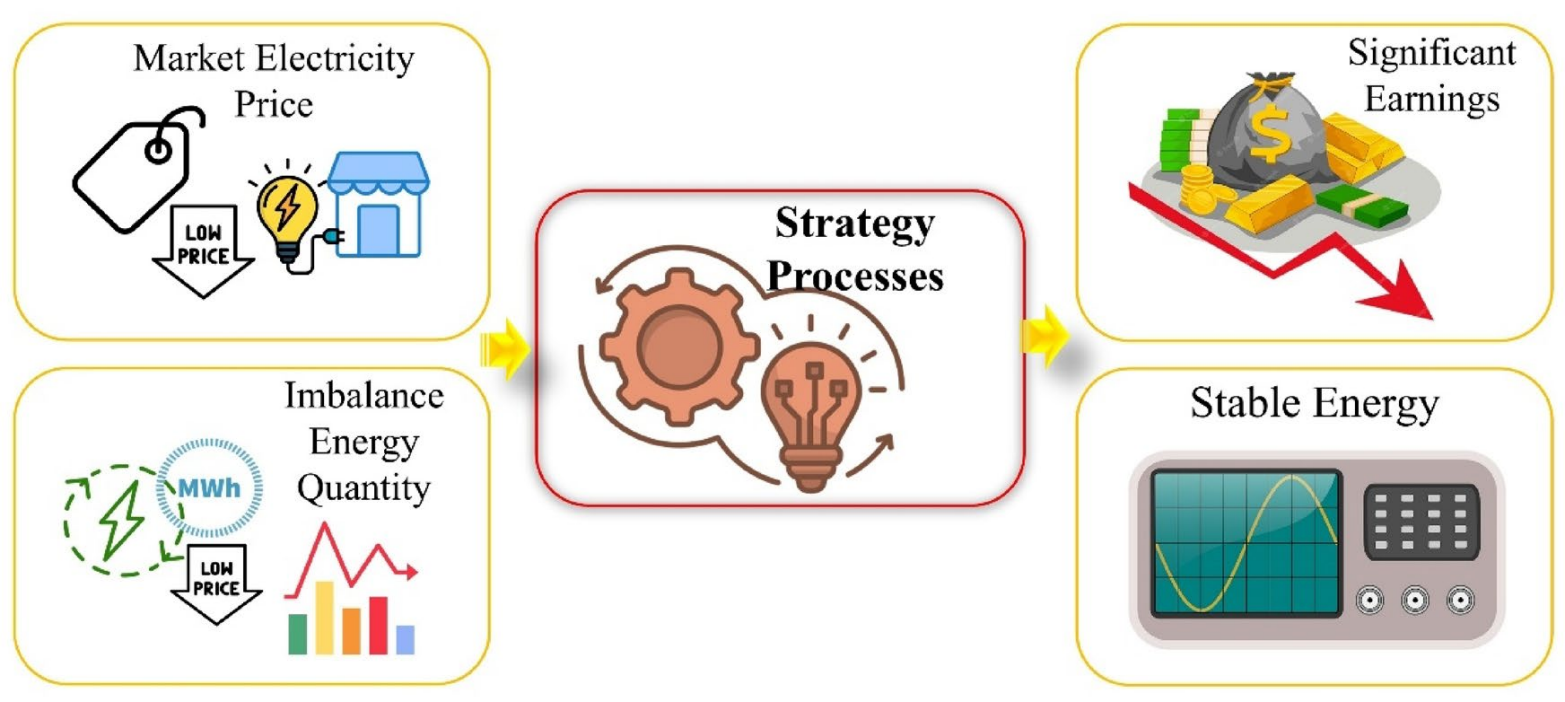
Schematic representation of the proposed strategy.

The strategies analyzed in the previous sections were focused either on power or price independently. However, real-time dynamics in the electrical grid may require multiple simultaneous actions during the day. When the amount of energy produced by a power plant is considered in parallel with electricity prices, situations may arise where smoothing the power at the plant’s output and achieving higher profitability can be realized simultaneously. This section develops a combined strategy, taking into account both imbalance and cost considerations, as illustrated in Fig. 8.

- $\:\text{M}\left(\text{t}\right)\:$ Imbalance level at time t (MW).
- $\:D\left(t\right)$ Energy amount to be discharged from the battery at time t (MW).
- $\:{\text{T}}_{16}$​, $\:{\text{T}}_{17}$​, $\:{\text{T}}_{18}$​ Threshold levels for imbalance (USD/hr).

**Fig. 9:**
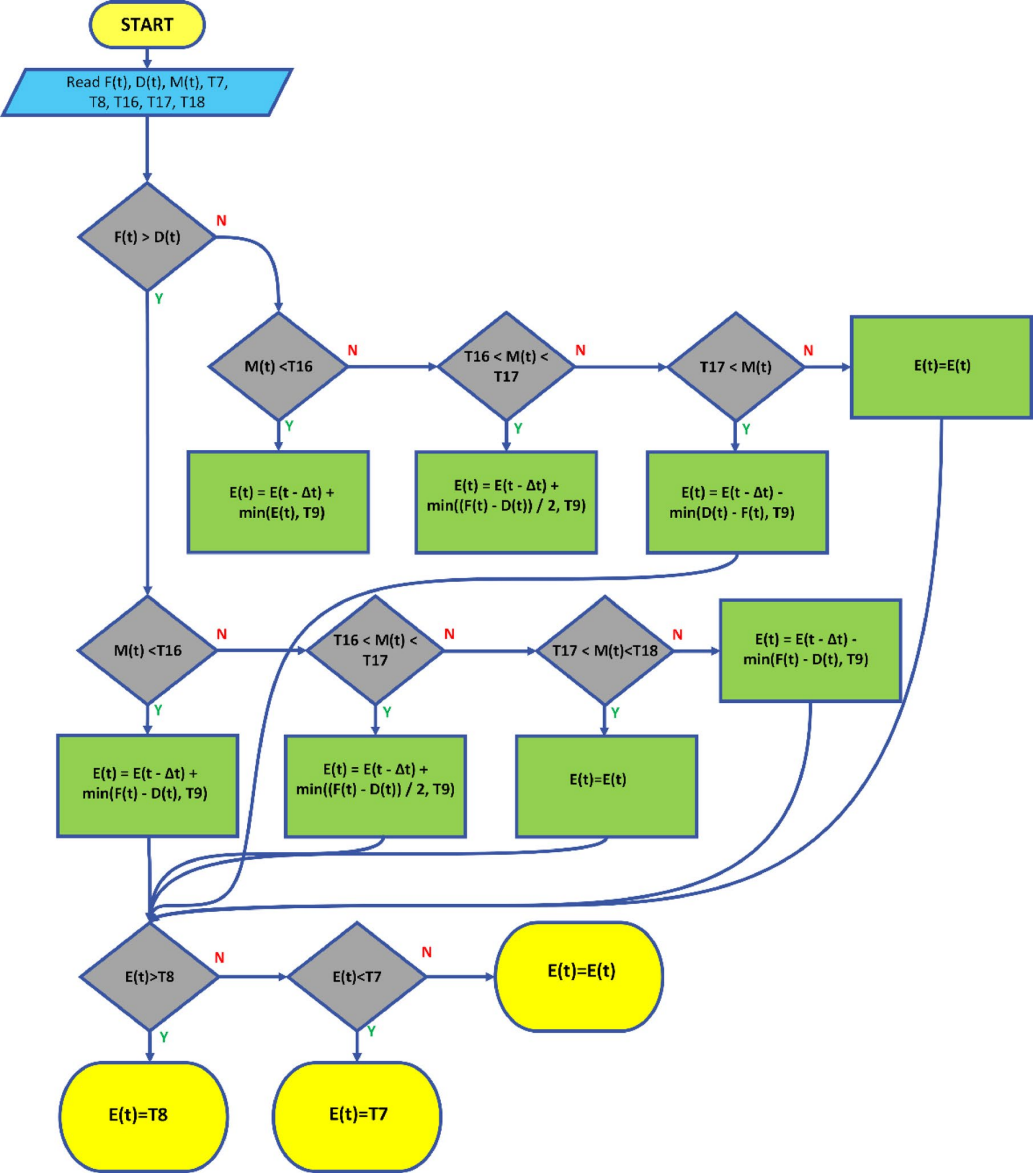
The flowchart of the proposed strategy.

If $\:\text{M}\left(t\right)$ is below the threshold $\:{\text{T}}_{16}$​, energy needs to be added to the battery. In this case, the current energy level $\:E\left(t-{\Delta\:}t\right)$ is compared with the minimum amount of energy to be added, and the smaller value is selected. If the energy to be added exceeds the $\:{\text{T}}_{9}$ limit, only $\:{\text{T}}_{9}$​ energy is added.

If $\:\text{M}\left(t\right)$ is above $\:{\text{T}}_{16}$​ and below $\:{\text{T}}_{17}$​, the amount of energy to be added to the battery is limited to $\:\frac{F\left(t\right)-D\left(t\right)}{2}$. In this case, the amount of energy to be added is compared with the $\:{\text{T}}_{9}$​ limit, and the smaller value is selected.

If $\:\text{M}\left(t\right)$ is above $\:{\text{T}}_{17}$​ and below $\:{\text{T}}_{18}$​, the battery’s energy level is not changed. In this case, the battery’s energy level remains at its current value $\:E\left(t-{\Delta\:}t\right)$.

If $\:\text{M}\left(t\right)$ exceeds $\:{\text{T}}_{18}$​ or in other cases, energy needs to be extracted from the battery. In this case, the current energy level $\:E\left(t-{\Delta\:}t\right)\:$is compared with the minimum amount of energy to be extracted, and the smaller value is selected. If the energy to be extracted exceeds the $\:{\text{T}}_{9}$​ limit, only $\:{\text{T}}_{9}$​ energy is extracted.

The flow diagram of the proposed strategy is illustrated in Fig. 9.

### Proposed operational strategy based on imbalance price and market price

**Fig. 10:**
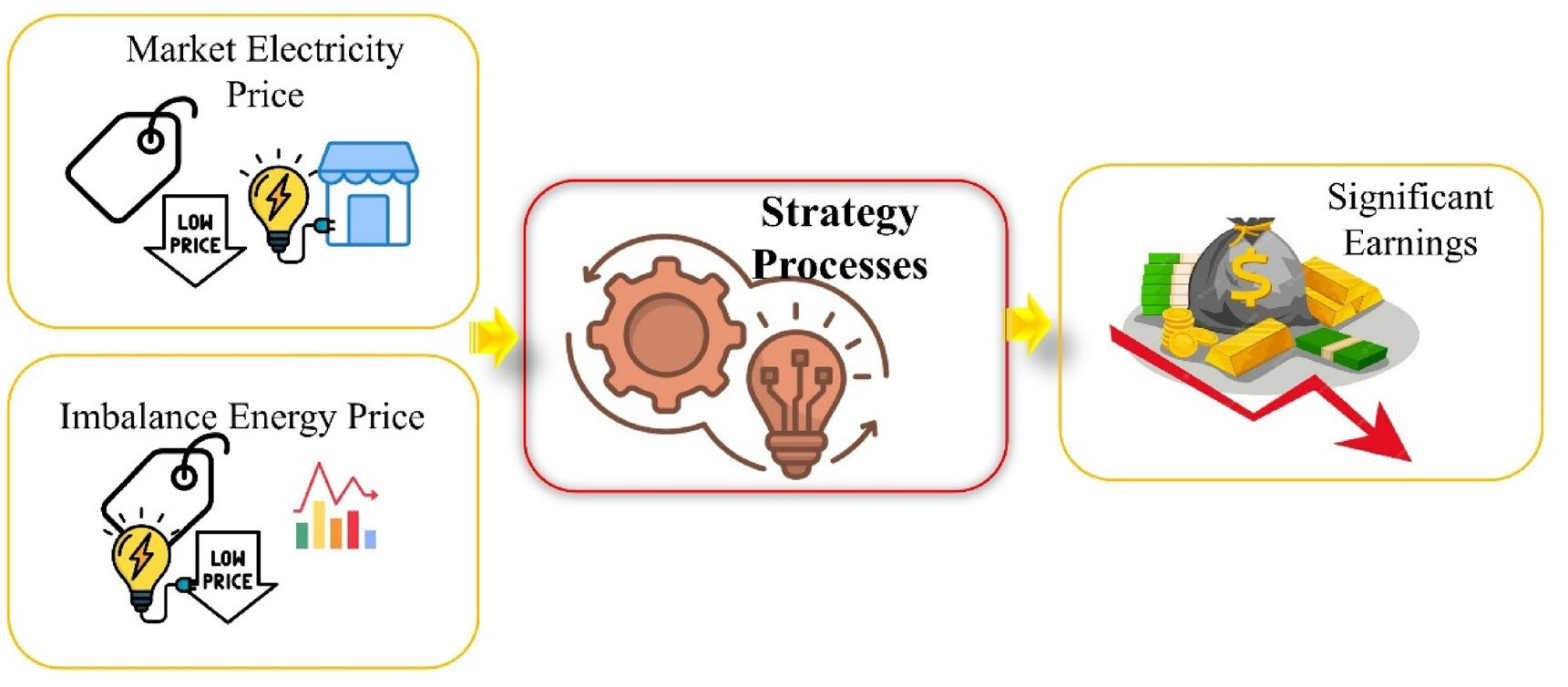
Schematic representation of the proposed strategy.

 In this strategy, instead of focusing solely on energy as analyzed in Sect. 2.3, the system remains unchanged, and only the price equivalent of the energy related components is incorporated into the equations. To explain more clearly, the imbalance MWh encountered during power smoothing is monetized, and a joint cost calculation is made by integrating this value with the market price. Therefore, a price oriented strategy has been developed under this section, as illustrated in Fig. 10.

11 $$\:E\left(t\right)=max\left.\left\{{\text{T}}_{7}\text{},min\left\{{\text{T}}_{8},\left\{\begin{array}{c}E\left(\text{t}-{\Delta\:}\text{t}\right)+min\left(F\left(t\right)-D\left(t\right),{\text{T}}_{9}\right),\:\:\:\:\:\:\:\:\:\:\:{\text{T}}_{25}<K\\\:E\left(\text{t}-{\Delta\:}\text{t}\right)+min\left(\frac{F\left(t\right)-D\left(t\right)}{2},{\text{T}}_{9}\right),\:\:\:\:\:\:\:\:\:\:\:F\:>D,M\\\:E\left(\text{t}-{\Delta\:}\text{t}\right),{\:\:\:\:\:\:\:\:\:\:\:\:\:\:\:\:\:\:\:\:\:\:\:\:\:\:\:\:\:\:\:\:\:\:\:\:\:\:\:\:\text{T}}_{16}\:\le\:\:M<{\text{T}}_{17}\\\:E\left(\text{t}-{\Delta\:}\text{t}\right)-min\left(F\left(t\right)-D\left(t\right),{\text{T}}_{9}\right)\:\:,\:\:otherwise\end{array}\right.\right\}\right\}\right\}$$

- $\:{\text{T}}_{25}$​ Threshold value for imbalance without a battery (USD/hr).
- $\:K$ Imbalance amount without a battery (MWh).
- $\:F\:\:$ Production amount without a battery (MWh).
- $\:M$ Profit amount without a battery (USD/hr).
- $\:D$ Scheduled production plan (MWh).
- $\:H$ Market electricity price (USD/hr).

**Fig. 11:**
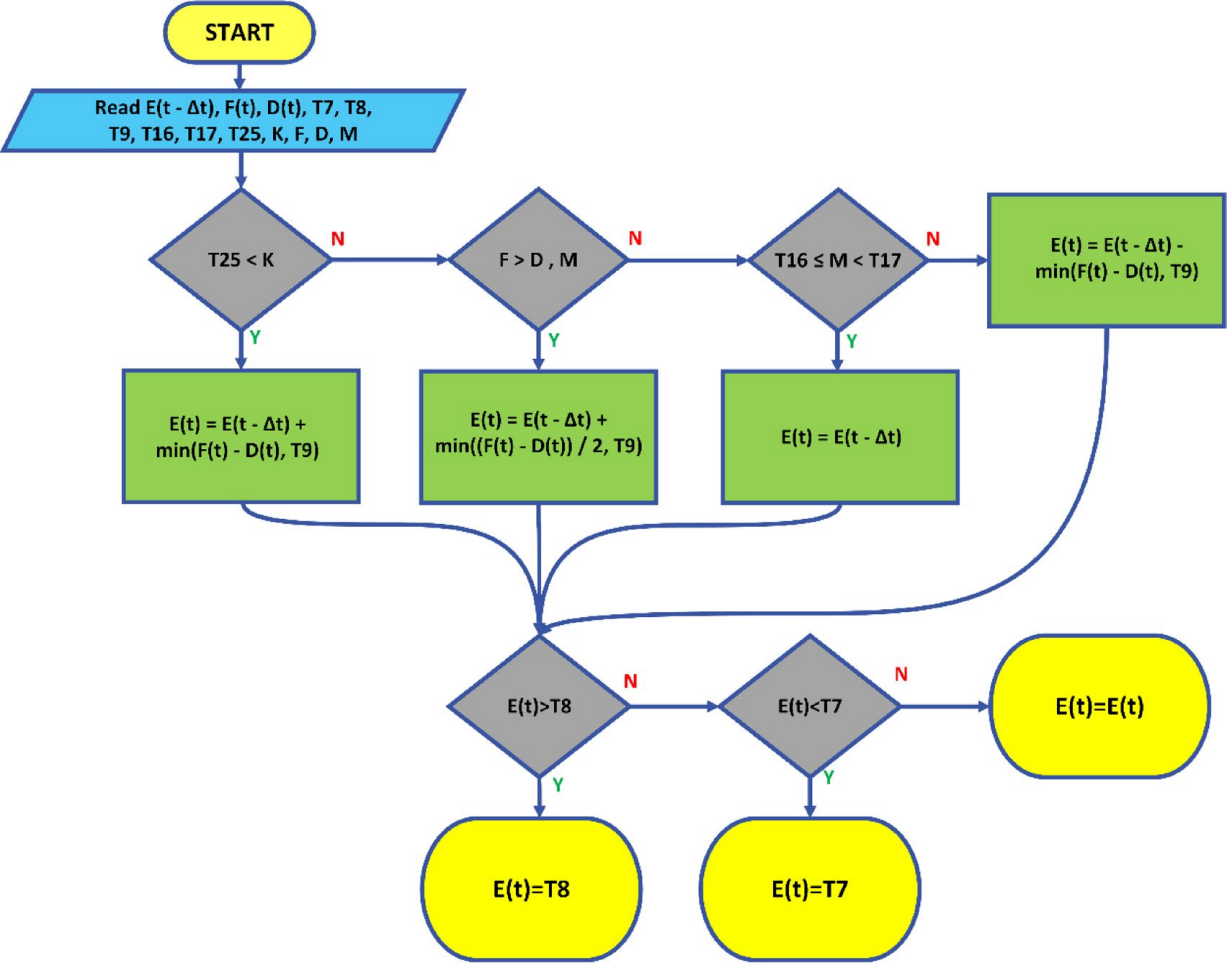
The flowchart of the proposed strategy.

Initially, the imbalance value without a battery ($\:K$) is compared with the imbalance threshold ($\:{\text{T}}_{25}$​). If this value is lower than the threshold, the battery’s energy level is updated as shown in Eq. 12.

12 $$\:E\left(t\right)=E(t-\varDelta\:t)+min\left(F\right(t)-D(t),{\text{T}}_{9})$$

In this case, the amount of energy added or removed from the battery is limited to a specific threshold ($\:{\text{T}}_{9}$​).

If the production amount without a battery ($\:F$) exceeds the imbalance ($\:K$) and the profit amount without a battery ($\:M$) is below a certain imbalance threshold ($\:{\text{T}}_{16}$​), the battery’s energy level is updated as shown in Eq. 13.

13 $$\:E\left(t\right)=E(t-\varDelta\:t)+min(\frac{F\left(t\right)-D\left(t\right)}{2},{\text{T}}_{9})$$

In this scenario, the amount of energy added or removed from the battery is restricted to less than half of the $\:F\left(t\right)-D\left(t\right)$ value.

If the profit amount without a battery ($\:M$) lies between two thresholds $\:{\text{T}}_{16}$​ and $\:{\text{T}}_{17\:}$, the battery’s energy level remains unchanged throughout the time step, maintaining its current level $\:E\left(t-\varDelta\:t\right)$.

14 $$\:E\left(t\right)=E\left(t-\varDelta\:t\right)$$

If the profit amount without a battery ($\:M$) exceeds $\:{\text{T}}_{17\:}$​, the battery’s energy level is updated as shown in Eq. 15.

15 $$\:E\left(t\right)=E(t-\varDelta\:t)-min\left(F\right(t)-D(t),{\text{T}}_{9})$$

In this case, the amount of energy added or removed can take on a negative value. The flowchart for this strategy is shown in Fig. 11.

### Proposed operational strategy for reducing imbalance price with a market and imbalance price-based approach

**Fig. 12:**
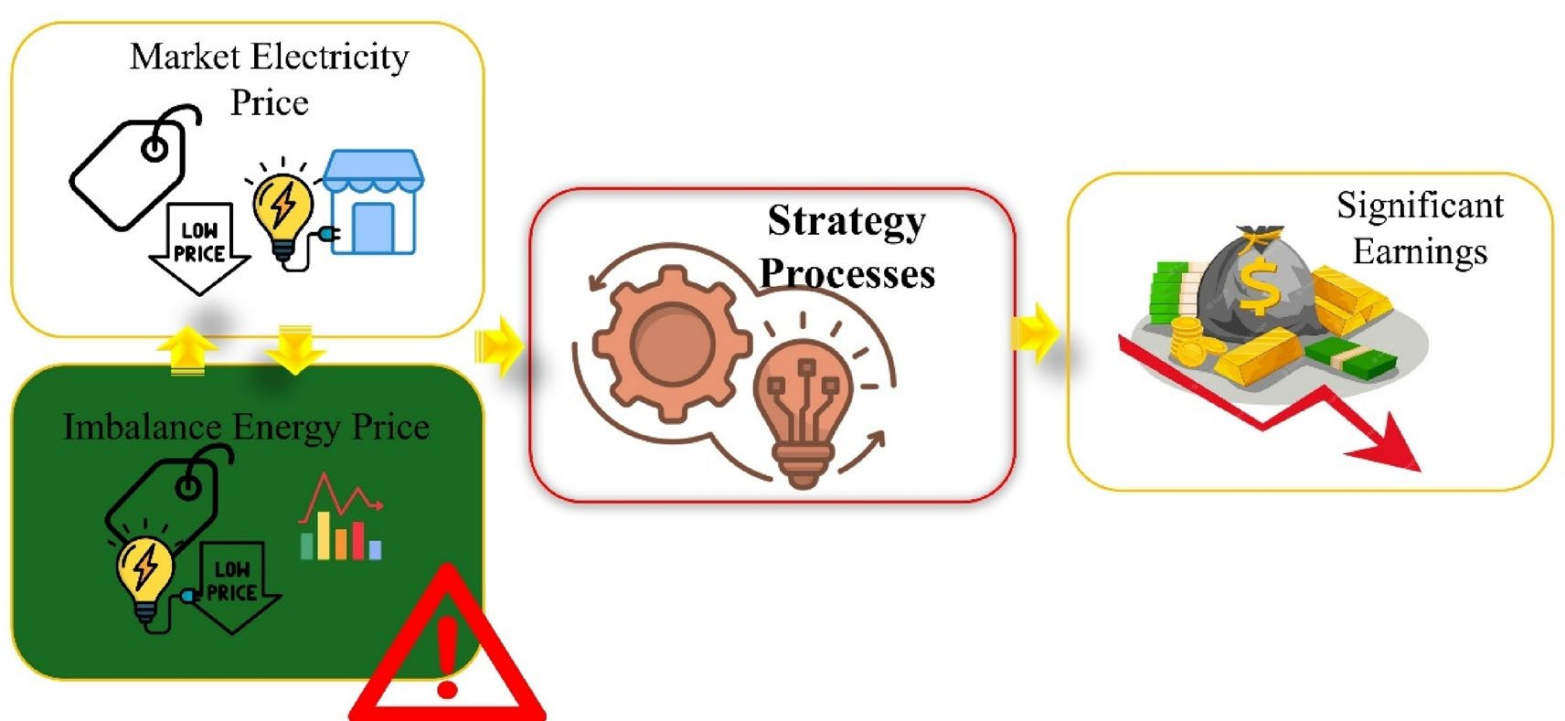
Schematic representation of the proposed strategy.

This strategy, developed as a cost focused approach, aims to maximize overall profitability based on insights from previous strategies. By coordinating the imbalance price and market price, this strategy prioritizes reducing imbalance as the primary objective while incorporating market price dynamics at critical points of sensitivity. Initially, power smoothing is performed according to the target prices. When limits are exceeded, market prices are integrated to create a balanced approach, aiming to maximize overall profitability. This scenario is illustrated in Fig. 12.

16 $$\:E\left(t\right)=min\left.\left\{{T}_{8}\text{},min\left\{{T}_{7},\left\{\begin{array}{c}E\left(t-\varDelta\:t\right)-min\left(F\left(t\right)-D\left(t\right),{T}_{9}\right),\:\:\:\:\:\:\:\:\:\:\:\:\:\:\:\:\:\:\:\:\:\:\:\:\:\:\:\:\:\:\:\:\:\:\:\:\:{T}_{27}<K\:ve\:F<D\\\:E\left(t-\varDelta\:t\right)+min\left(F\left(t\right)-D\left(t\right),{T}_{9}\right),\:\:\:\:\:\:\:\:\:\:\:\:\:\:\:\:\:\:\:\:\:\:\:\:\:\:\:\:\:\:\:\:\:\:\:\:\:{T}_{27}<K\:ve\:F<D\\\:\left\{\begin{array}{c}E\left(t-\varDelta\:t\right)+min\left(F\left(t\right)-D\left(t\right),{T}_{9}\right),\:\:\:\:\:\:\:\:\:\:\:{T}_{25}<K\:ve\:\:F>D\:ve\:{T}_{16}>M\\\:E\left(t-\varDelta\:t\right)+min\left(\frac{F\left(t\right)-D\left(t\right)}{2},{T}_{9}\right),\:{T}_{25}<K\:ve\:\:F>D\:ve\:{T}_{16}<M<{T}_{17}\\\:E\left(t-\varDelta\:t\right),\:\:\:\:\:\:\:\:\:\:\:\:\:\:\:\:\:\:\:\:\:\:\:\:\:\:\:\:\:\:\:\:\:\:\:\:\:\:\:\:\:\:\:\:\:\:\:\:{T}_{25}<K\:ve\:\:F>D\:ve\:{T}_{17}<M<{T}_{18}\\\:E\left(t-\varDelta\:t\right)-min\left(F\left(t\right)-D\left(t\right),{T}_{9}\right),\:\:\:\:\:\:\:\:\:\:\:\:\:\:{T}_{25}<K\:ve\:\:F>D\:ve\:M>{T}_{18}\end{array}\right.\\\:\left\{\begin{array}{c}E\left(t-\varDelta\:t\right)+min\left(\text{F},{T}_{9}\right),\:\:\:\:\:\:\:\:\:\:\:\:\:\:\:\:\:\:\:\:\:\:\:\:\:\:\:\:\:\:\:\:\:\:\:\:\:\:\:\:\:\:\:\:\:\:\:\:\:\:\:\:\:\:\:\:\:\:\:\:\:\:\:\:\:\:\:\:\:\:\:\:\:\:\:\:\:H<{T}_{14}\\\:E\left(t-\varDelta\:t\right)-min\left({T}_{9},E\left(t-\varDelta\:t\right)\right),\:\:\:\:\:\:\:\:\:\:\:\:\:\:\:\:\:\:\:\:\:\:\:\:\:\:\:\:\:\:\:\:\:\:\:\:\:\:\:\:\:\:\:\:\:\:\:\:\:\:\:\:\:\:H>{T}_{15},{T}_{12}\\\:E\left(t-\varDelta\:t\right),\:\:\:\:\:\:\:\:\:\:\:\:\:\:\:\:\:\:\:\:\:\:\:\:\:\:\:\:\:\:\:\:\:\:\:\:\:\:\:\:\:\:\:\:\:\:\:\:\:\:\:\:\:\:\:\:\:\:\:\:\:\:\:\:\:\:\:\:\:\:\:\:\:\:\:\:\:\:\:\:\:\:\:\:\:\:\:\:\:\:\:\:\:\:\:\:\:otherwise\end{array}\right.\end{array}\right.\right\}\right\}\right\}$$

**Fig. 13:**
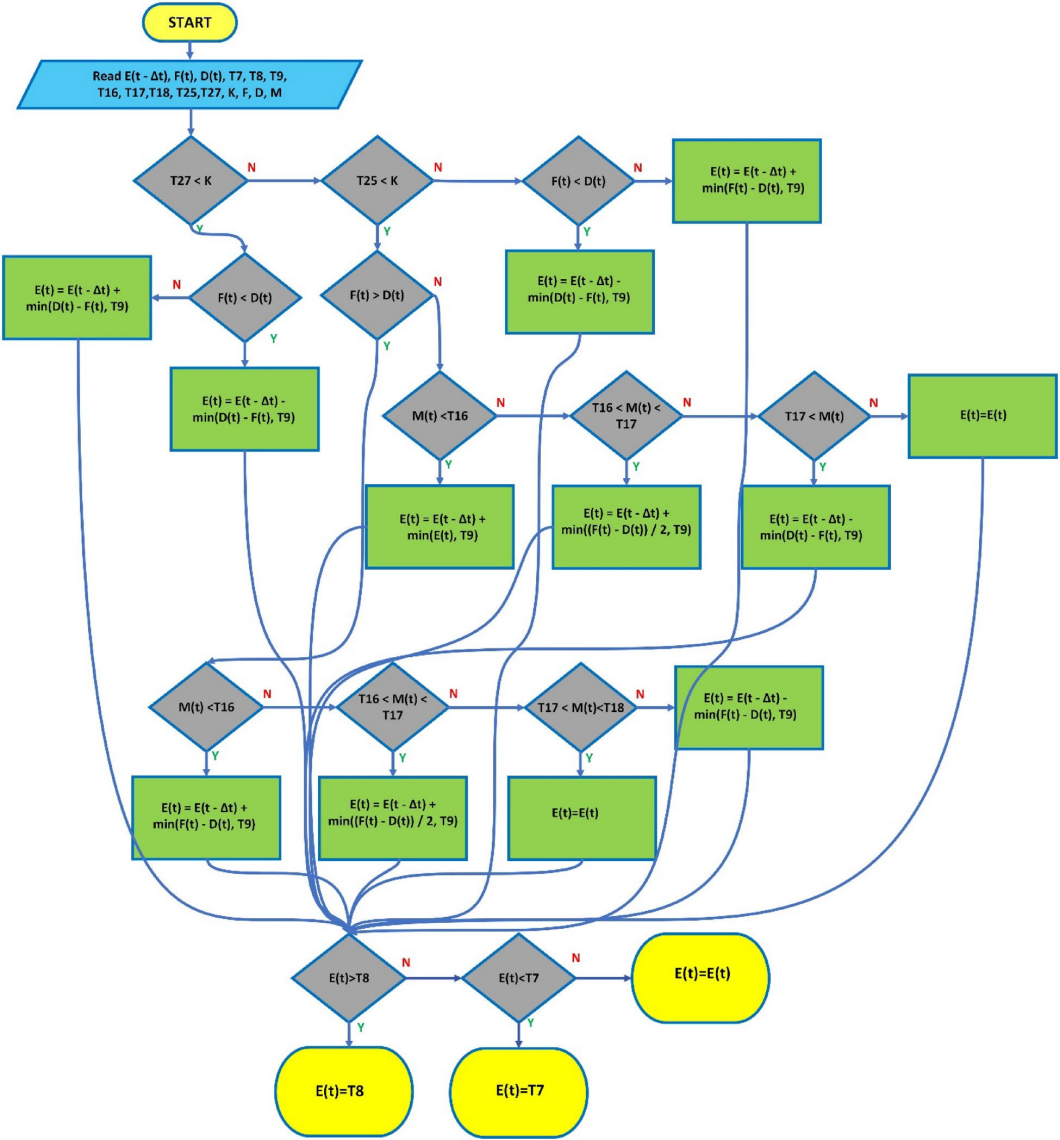
The flowchart of the proposed strategy.

Initially, the imbalance amount without a battery ($\:K$) is compared with the imbalance threshold ($\:{\text{T}}_{25}$​). If this value is below the threshold, the battery’s energy level is updated accordingly. If the production amount without a battery ($\:F$) is less than the imbalance ($\:K$) and the profit amount without a battery ($\:M$) is also below the imbalance, the battery’s energy level is updated as shown in Eq. 17.

17 $$\:E\left(t\right)=E(t-\varDelta\:t)-min\left(D\right(t)-F(t),{\text{T}}_{9})$$

In this case, the amount of energy extracted from the battery is limited to a specific threshold ($\:{\text{T}}_{9}$​). If the production amount without a battery ($\:F$) exceeds the imbalance ($\:K$) and the profit amount without a battery ($\:M$) is below a certain threshold ($\:{\text{T}}_{16}$​), the battery’s energy level is updated as shown in Eq. 18.

18 $$\:E\left(t\right)=E(t-\varDelta\:t)+min\left(F\right(t)-D(t),T9)$$

In this case, the amount of energy added to the battery is limited to a specific threshold ($\:{\text{T}}_{9}$​).

If the production amount without a battery ($\:F$) exceeds the imbalance ($\:K$) and the profit amount without a battery ($\:M$) is greater than a certain threshold ($\:{\text{T}}_{16}$​), the battery’s energy level is updated as shown in Eq. 19.

19 $$\:E\left(t\right)=E(t-\varDelta\:t)+min(\frac{\left(F\right(t)-D(t)}{2},T9)$$

In this case, the amount of energy added to the battery is limited to a specific threshold ($\:{\text{T}}_{9}$​), but this value is adjusted based on the imbalance threshold.

In other cases, where the production amount without a battery ($\:F$) equals the imbalance value ($\:K$) or the profit amount without a battery ($\:M$) does not exceed the threshold ($\:{\text{T}}_{16}$​), the battery’s energy level is updated as shown in Eq. 20.

20 $$\:E\left(t\right)=E(t-\varDelta\:t)$$

In this scenario, the battery’s energy level remains constant, with no charging or discharging performed. The flowchart for this strategy is shown in Fig. 13.

All the developed strategies aim to enhance the efficiency of hybrid facilities. However, an important consideration that should not be overlooked is determining the appropriate battery capacity. Since battery capacity is a fixed value, it directly affects not only the performance of the facility but also the entire strategic process. If the capacity is miscalculated, the facility’s efficiency may decrease, and the desired outcomes may not be achieved, no matter how effective the strategies are.

Determining the correct battery capacity ensures optimized energy production and storage processes. Furthermore, it enhances the facility’s resilience to future energy demand increases or sudden fluctuations. In conclusion, selecting the right battery capacity maximizes the effectiveness of the strategies, contributing to the long term sustainability and success of hybrid facilities.

## Battery A capacity configuration

The capacity configuration of a battery facility requires a careful planning and analysis process. As a first step, the intended purpose of the facility and its storage needs must be comprehensively analyzed. Storage capacity and power requirements should be determined in alignment with the expected usage scenarios. During this process, various energy storage technologies, such as lithium-ion batteries, should be carefully evaluated in terms of cost, energy density, and lifespan.

The configuration and connection of batteries must align with the facility’s voltage and energy storage requirements. Additionally, safety and environmental factors hold significant importance; particularly waste management and fire risks must be considered^63,64^. Furthermore, adherence to local regulations and international standards is critical. Depending on the specific objectives of the facility, additional functions such as providing backup power or improving power quality should also be assessed.

For the efficient operation of a battery facility, regular maintenance and monitoring processes must be planned^65^. All steps of the project should be tailored to specific requirements. At this stage, linear programming techniques come into play. Linear programming offers a robust tool for addressing the numerous variables and constraints encountered in battery capacity configuration. The literature includes numerous studies demonstrating the effective use of this method in optimizing complex energy systems. For instance, Hüseyin Abdellatif and colleagues employed linear programming to optimize complex structures in renewable energy systems for multiple objectives^66^. Similarly, Yujian Zhang, Mingde Li, and Fei Tong contributed to the literature by using linear programming to improve energy efficiency and load profile management in their studies^67^.

In this context, choosing the linear programming method for the capacity configuration of a battery facility can be considered a rational step to enhance operational efficiency and optimize costs. By utilizing linear programming, energy storage systems within the facility can be optimally configured, thus achieving the best outcomes both economically and technically.

### Optimal capacity configuration using linear programming

The proposed mathematical model, based on the linear programming technique, is designed to determine the battery’s energy capacity and identify the most cost effective value. The purpose of this design is to find the optimal capacity value for a given investment.

In this study, a deterministic optimization framework was adopted to evaluate the integration strategies of wind power and battery energy storage. The rationale for this choice is twofold. First, the primary objective of the research is to compare and highlight the strategic value of different operation modes, for which a deterministic setting provides a transparent and tractable basis. Second, deterministic models enable clear benchmarking of strategies without the added complexity of stochastic uncertainty modeling. While we acknowledge that wind generation and demand inherently involve variability, incorporating stochastic or uncertainty aware methods is considered a promising direction for future research.

#### Objective function

In Eq. (7), we defined the expression $\:\sum\:_{t=0}^{t}\left(\beta\:\right(t)-minCA-\gamma\:)>0$.

To maximize this expression, the value of $\:\beta\:\left(t\right)$should reach its maximum, while the values of $\:minCA$ and $\:\gamma\:$ should be minimized. This forms the target we aim to achieve.

The expression $\:\beta\:\left(t\right)=E\left(t\right).C\left(t\right)$ in Eq. (4) and the expression

$$\:minCA=\frac{{r(1+r)}^{yA}}{{(1+r)}^{yA}-1}\cdot\:(Cbp\text{}\cdot\:PA+Cbe\text{}\cdot\:EA+Crb\text{}\cdot\:EA)+{C}_{LC}$$.

in Eq. (1) vary based on E, since E is the time-dependent variable of P, the power value we aim to achieve. For the time-dependent variation of P, the variable is P(t), and our objective function is expressed as:

21 $$\:max\:\:{f}_{t}\left\{\sum\:_{t=0}^{t}\left(\beta\:\right(t)-minCA-\gamma\:)\right\}\:\:,\:\:\:\:\:\:\:\:\:t=\text{1,2},\text{3,4}....tmax$$

​ 

#### Constraints

Target capacity value: $\:\text{P}\left(\text{m}\text{i}\text{n}\right)<\text{P}\left(\text{t}\right)\le\:100$

T: Fixed time period, $\:\text{}\:\text{t}=30\:\text{d}\text{a}\text{y}\text{s}$

#### Limitations

The storage efficiency was modeled as a constant value, which does not capture variations due to charging rate, discharging rate, or battery aging effects.

### Battery performance and lifespan

Battery performance and lifespan are critically important for the efficiency and operational continuity of these technologies. One of the most crucial parameters determining the condition and lifespan of batteries is the state of charge (SOC), which indicates the instantaneous energy level of the battery. SOC serves as a fundamental indicator for the effective management and optimization of batteries, providing significant information to both energy management systems and users. Accurate and reliable estimation of SOC is essential for extending battery life, improving energy efficiency, and ensuring safe operating conditions.

In energy systems, the importance of this parameter has started to become evident in studies. A strategy has been proposed in^68^ to regulate the response priorities of batteries in stepwise energy storage systems. This strategy optimizes the charging and discharging processes of batteries based on their health conditions. By enabling healthy batteries to undergo deep discharges and less healthy batteries to perform shallow discharges, it has been reported that this method improves battery lifespan and performance compared to traditional approaches. This approach reduces the cycling frequency and charge discharge switching of batteries, thereby increasing the efficiency of energy storage systems. Future research will focus on factors affecting the prioritization within this strategy.

In battery Management, Maintaining the SOC of batteries within the 20–80% range is critical to Maximizing their performance and Lifespan. Batteries operating within this range are exposed to less chemical and mechanical stress, allowing for more efficient energy conversion and longer durability. When SOC falls below 20% or exceeds 80%, batteries face risks of over discharge and overcharge, which can result in capacity loss and shortened lifespan. Studies in^20^ have evaluated maintaining the SOC within this optimal range as a strategic measure to enhance the overall efficiency of energy storage systems and prolong battery life.

Based on these studies, all strategies developed in Chap. 3 have been enhanced to incorporate SOC considerations in addition to the current conditions. In the results section, scenarios including SOC are presented alongside the current conditions. This allows for a comparison between the previous and enhanced states of a battery facility used in the energy sector.

## Case study

The impact of energy storage systems on wind energy production and the applicability of these systems have been exemplified in detail. The study was conducted using December 2023 data from a private wind energy plant in the Karacabey district of Bursa, Turkey, within the interconnected Turkish grid system. To ensure accurate comparisons, all analyses were performed for two different scenarios: one with battery systems and one without.

### Results of modeling under different strategies

The imbalance cost resulting from production in a scenario without batteries, i.e., when no battery system is integrated into the system (t = 0), is compared with the results of operational strategies developed under specific strategies with added battery systems. Strategies are presented according to their numbering, and for each strategy, the corresponding strategy including SOC is provided immediately afterward.

**Fig. 14:**
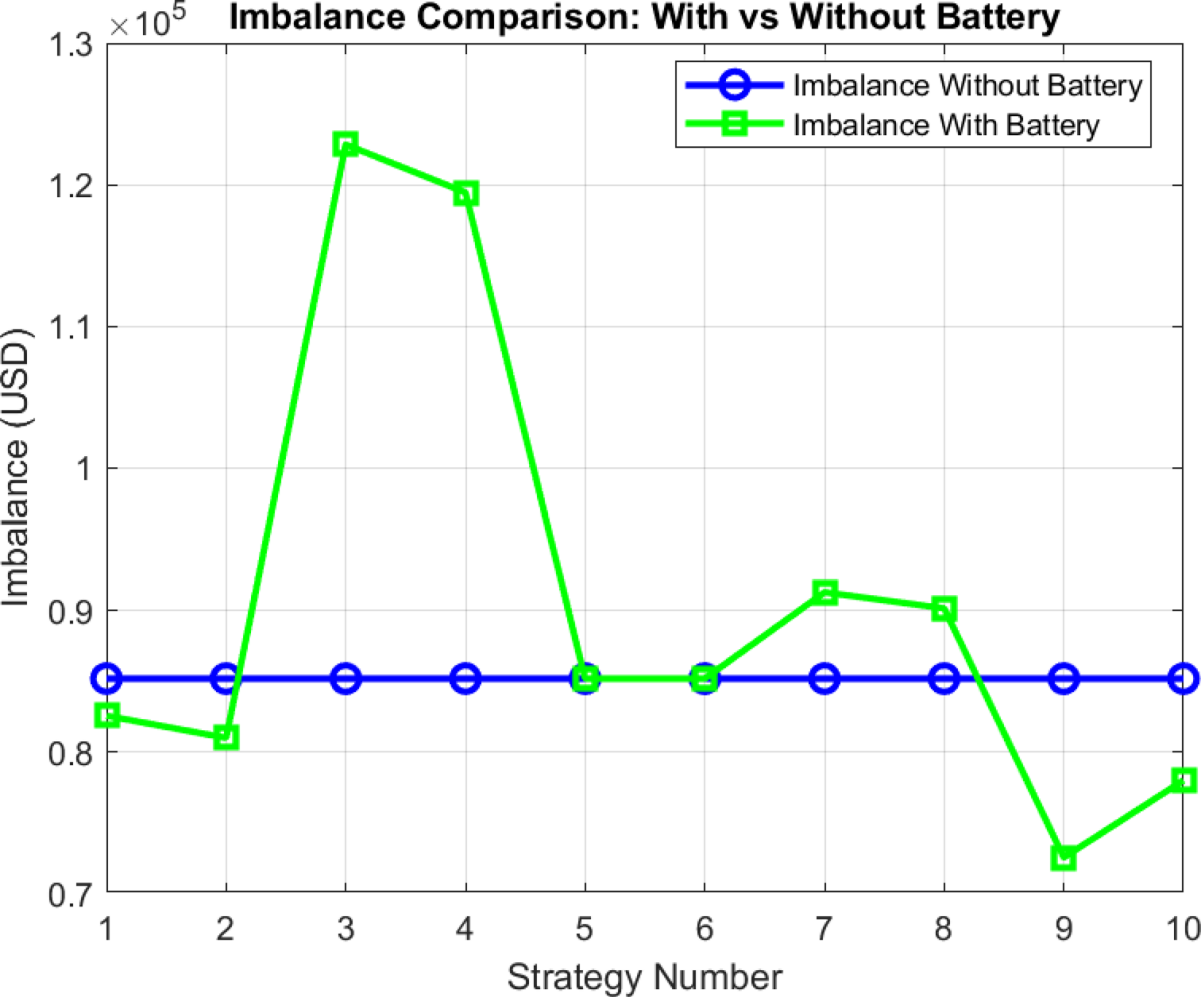
Analysis of the developed strategies based on imbalance price.

In Fig. 14, it is observed that the imbalance cost for strategies indexed as 3 and 4 is higher compared to the cost at t = 0. However, in all other strategies, the cost is observed to be lower, indicating improved outcomes.

For most businesses or investors, the primary factor considered is production revenue. Investors evaluate this data as the main income item in their investments. High production revenue is crucial both in terms of profitability and the return on investment. An increase in production revenue ensures higher investment returns, which positively influences investors’ decisions.

**Fig. 15:**
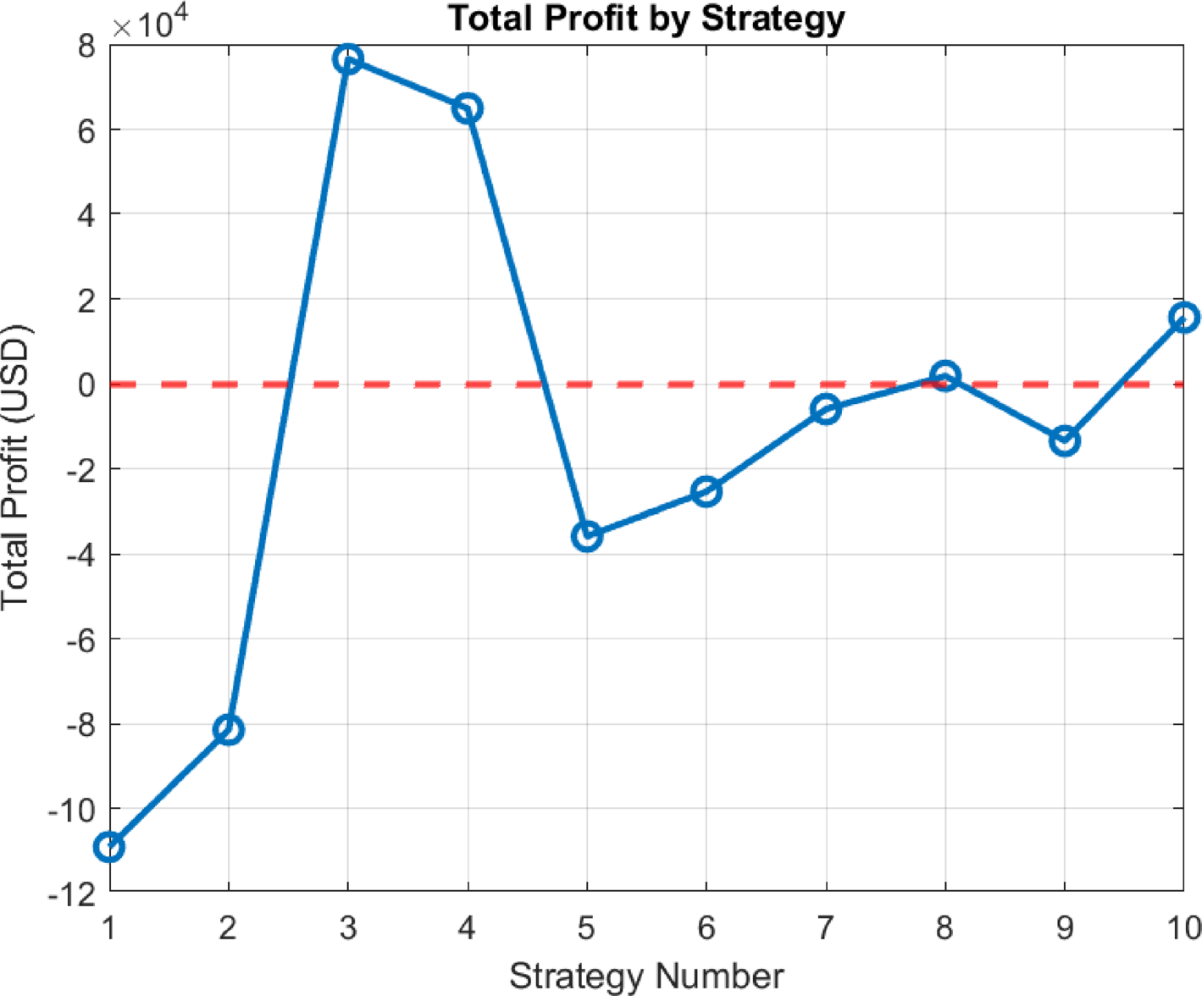
Analysis of the developed strategies based on production revenue.

Looking at the graph in Fig. 15, it is observed that, when the wind plant is supported with a battery system, strategies 3, 4, and 7 show positive changes in total revenue. In particular, strategies 3 and 4 demonstrate a more significant difference compared to other strategies. The increase in production revenue in these strategies clearly highlights the positive impact of battery integration on the efficiency of wind plants. Therefore, it can be said that battery-supported strategies make investments more attractive by shortening the return on investment period and increasing profitability.

**Fig. 16:**
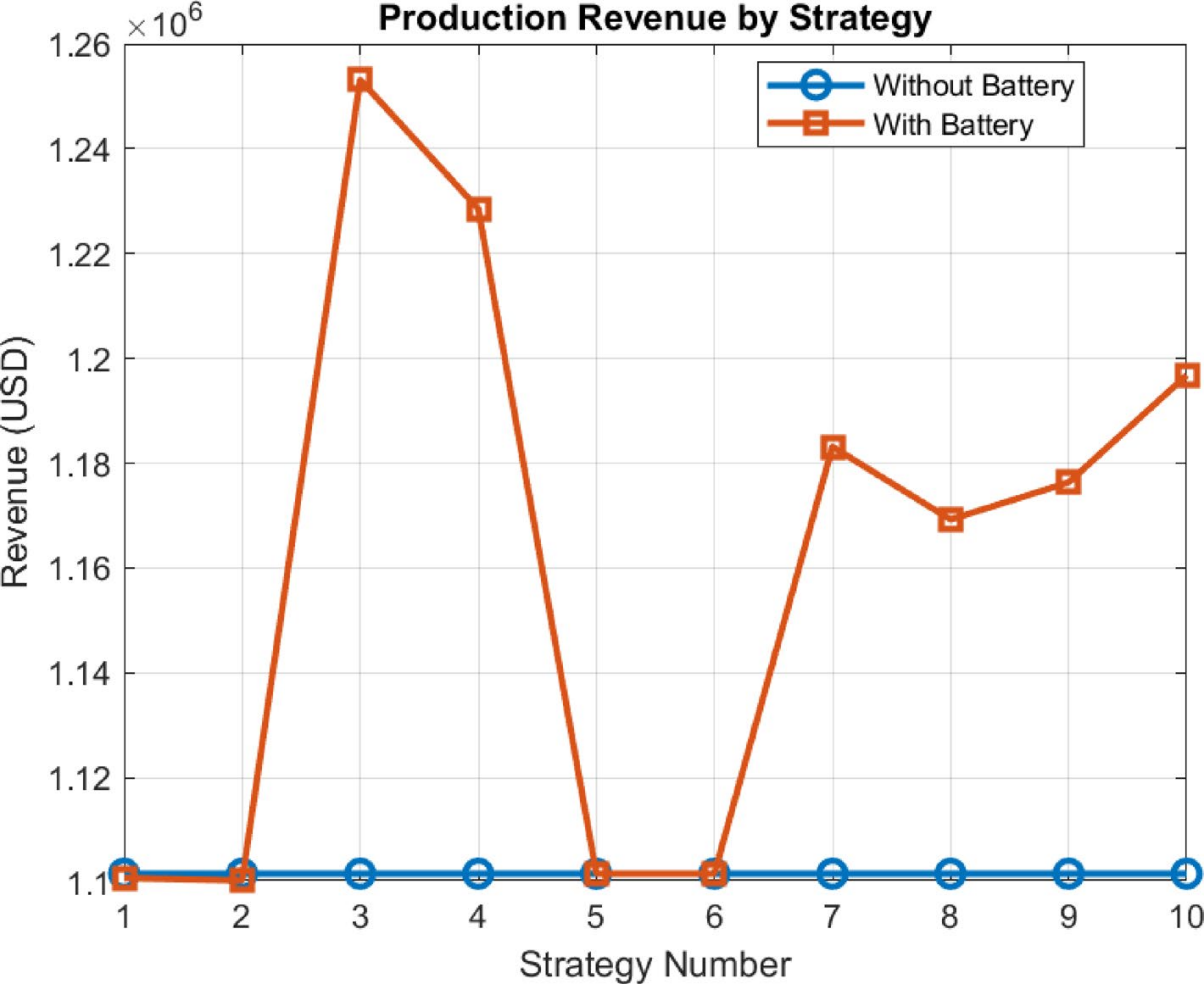
Total profit result.

When examining the situation presented in Fig. 16, a more concise and comprehensible overview is provided. This table considers investment costs and total conditions together. Investment costs have been reduced to a one month period. That is, the financial burden and return of the investment have been analyzed in monthly periods, providing a general evaluation in this Manner. Strategies 3 and 4 appear to be more profitable compared to the others.

### Results of capacity optimization configuration

**Fig. 17:**
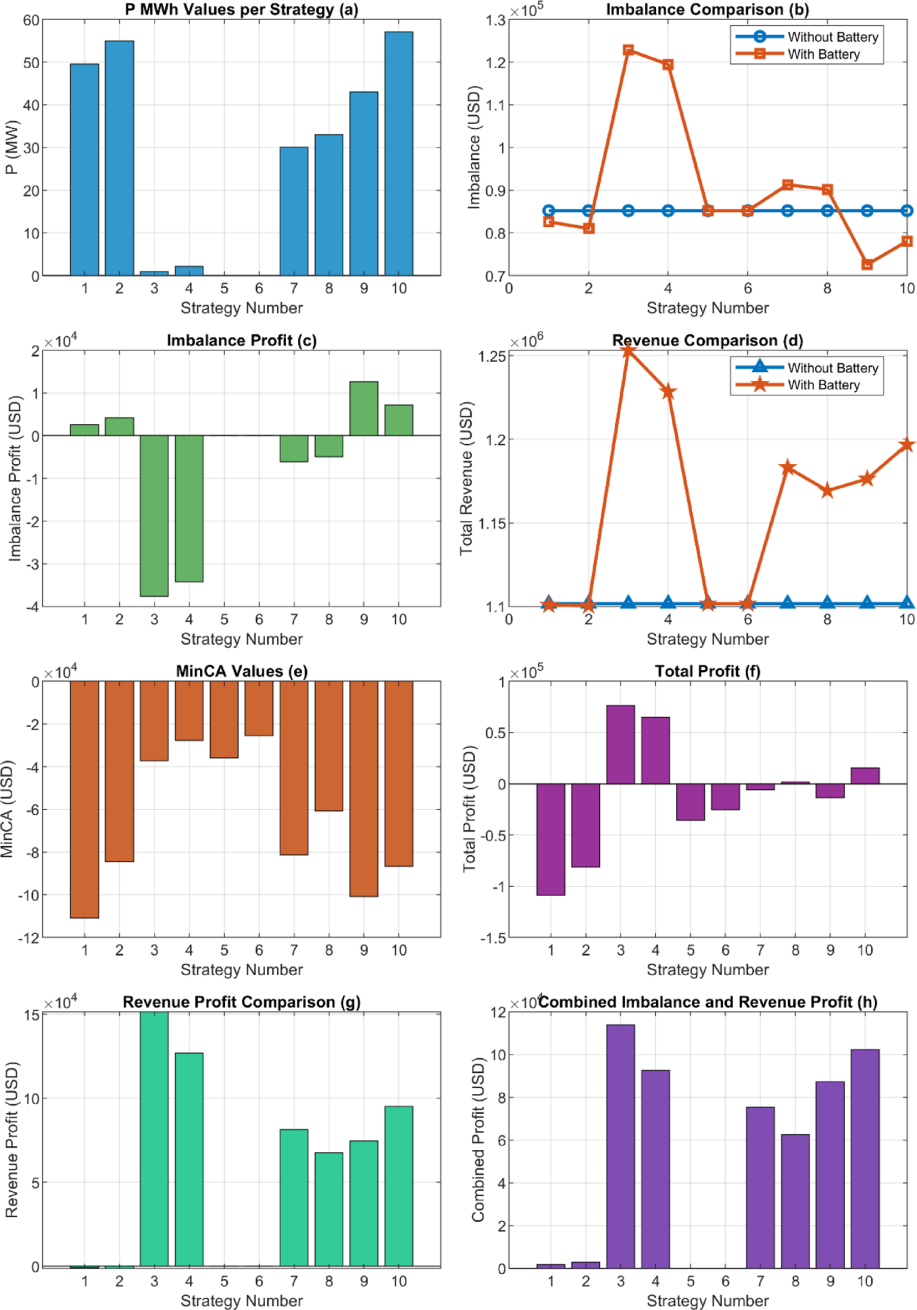
Results Obtained According to Strategies (**a**). P Optimization Results (**b**). Battery vs. Non-Battery Imbalance Cost (**c**). Imbalance Gain (**d**). Total Revenue Status (**e**). CAPEX Expenses (**f**). Total Profit (**g**). Revenue Gain (**h**). Total Imbalance and Revenue Gain Status.

 The developed strategies have been derived based on the results provided in Sect. 6.1. Using these results, the question of what capacity is required to maximize total profitability has been addressed by incorporating the optimization process into the equation. The fixed coefficients used in the equations are provided in Table 2. The results obtained from the optimization process are shown in Fig. 17.

**Table 2:** Constant coefficients used in optimization.

T14	Minimum Market Price	51,98	USD
T15	Maximum Market Price	73,33	USD
T16	Revenue Price 1	50,21	USD
T17	Revenue Price 2	106,19	USD
T18	Revenue Price 3	117,37	USD
T25	Imbalance Price 1	151,51	USD
T27	Imbalance Price 2	303,03	USD

The results in Fig. 17 show the variation of optimized P values according to energy strategies and the total profits. While the “Operational Strategy Based on Market Electricity Price” achieves the highest P value, the “Strategy Considering Imbalance MWh Amount and Market Price Together” provides a P value close to zero. The total profits of the strategies also vary, with the “Operational Strategy Based on Market Electricity Price” standing out among the most profitable ones. Other strategies demonstrate lower profit values. Additionally, it is observed that the SOC-inclusive versions perform better than the standard strategies.

The results obtained in Fig. 17 are analyzed in detail under the following headings:

#### Analysis of P optimization values based on energy strategies

The variation in P (MW) values among strategies clearly reflects the impact of these strategies on the energy system. P represents the fluctuating energy quantity in the energy system and indicates how the strategy manages this fluctuating energy. A lower P value suggests that the strategy more effectively controls energy imbalances and adapts better to market conditions, whereas a higher P value indicates greater imbalances and more significant energy deviations under the strategy.

**Fig. 18:**
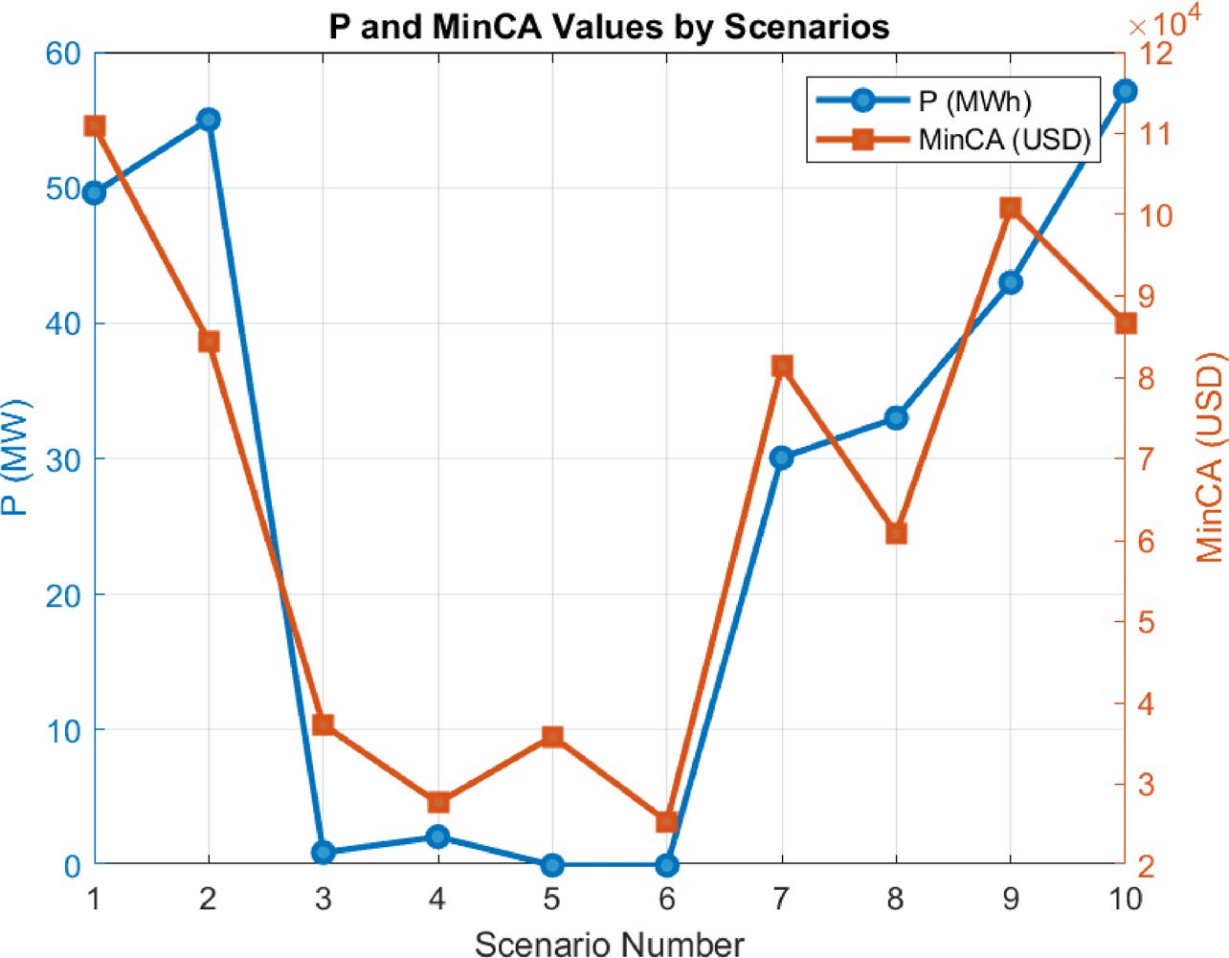
P optimization values formed according to strategies and the corresponding investment cost.

For certain strategies (e.g., 3.1), the P optimization value is higher, while for others (e.g., 3.3), it is close to zero. This demonstrates that strategies optimize energy balance differently depending on their specific objectives and market prices. Strategies designed based on market electricity prices may be less effective in reducing energy imbalances caused by price fluctuations, whereas those focusing on imbalance prices manage fluctuating energy more efficiently. The magnitude of P also directly affects initial investment costs. As shown in Fig. 18, changes in P influence investment costs as well.

In this context, the differentiation of P values serves as a critical indicator for evaluating the flexibility of energy management strategies and their ability to adapt to the market.

#### Impact of strategies on energy imbalance

**Fig. 19:**
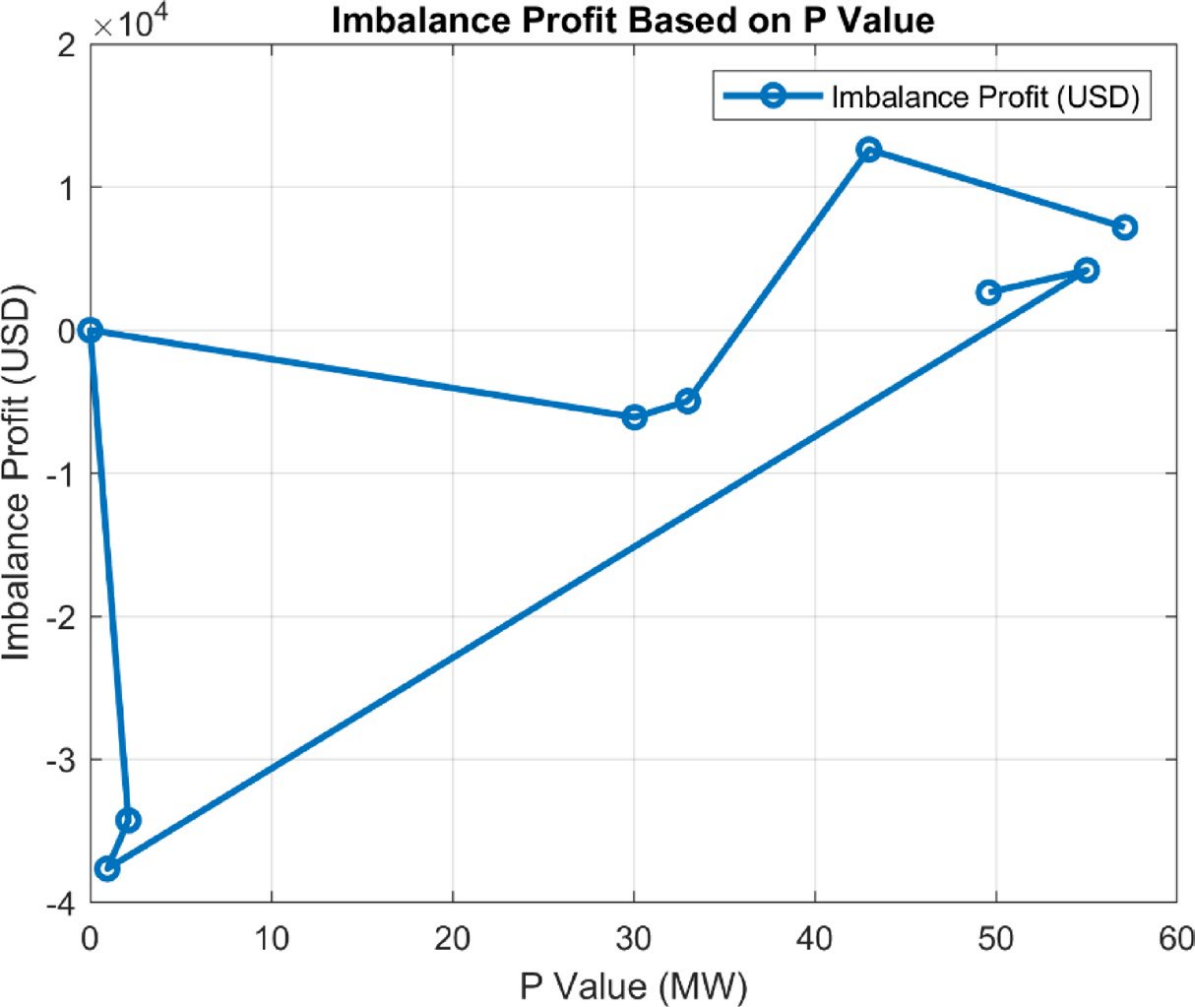
P Optimization values and the corresponding imbalance gains.

Each strategy has managed energy imbalances differently, leading to variations in P (MW) optimization values. Minimizing imbalance has been a significant indicator of the efficiency of strategies. For market price based strategies (e.g., 3.3 and 3.4), insufficient attention to imbalances has resulted in losses ranging from 30,000 USD to 40,000 USD. In contrast, imbalance-sensitive strategies have achieved positive results. This finding suggests that such strategies should be preferred in cases where controlling market imbalances is critical.

Since the strategies were not created based on a particular flow or system, it is expected that the results in the graphs are neither parabolic nor linear. This can be clearly observed in Fig. 19, where the outcomes vary entirely depending on the scenario of strategy development. For instance, the highest imbalance profit, achieved under the strategy examined in **3.9**, was obtained with a 42.8 MW battery capacity.

**Fig. 20:**
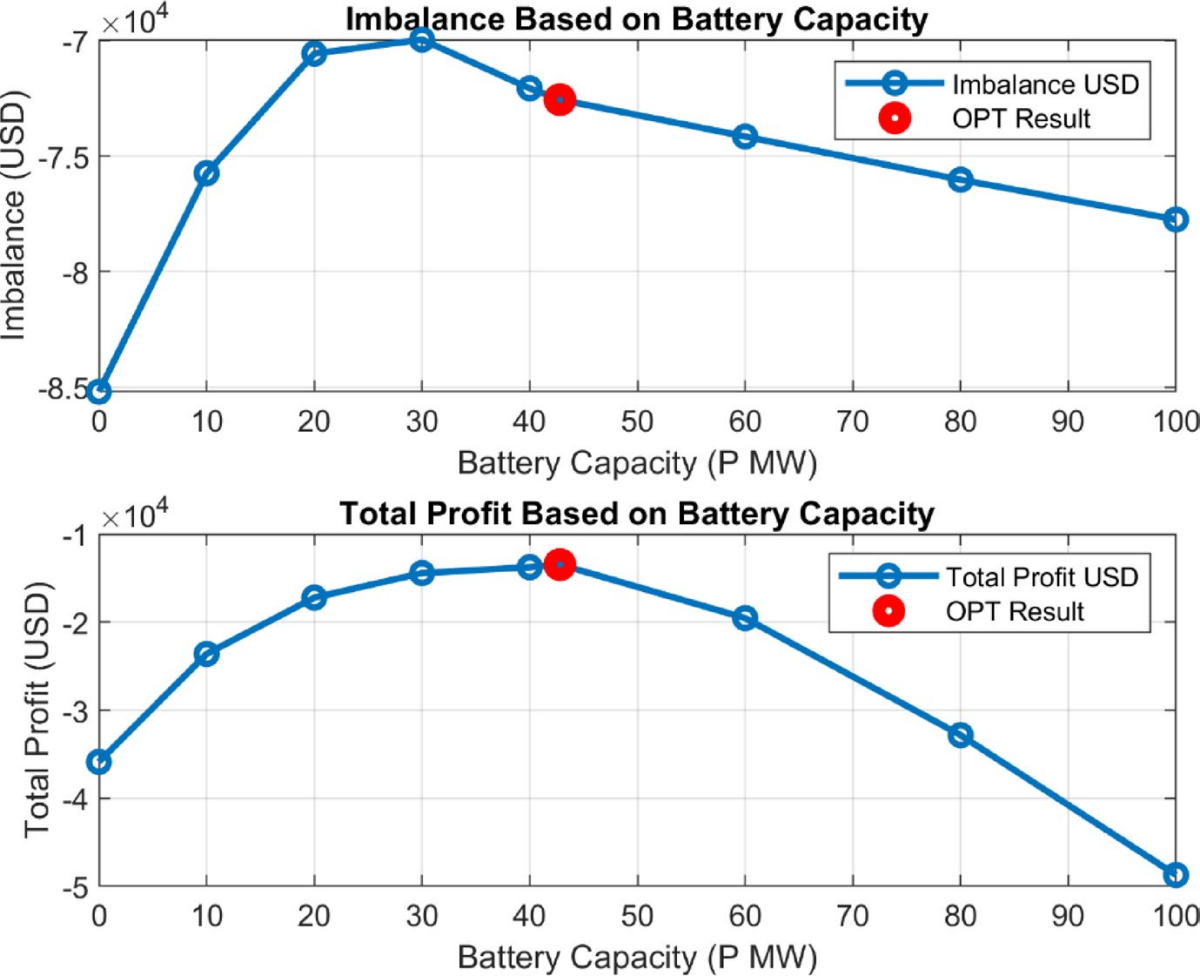
Imbalance and total gain corresponding to the 0-100 MW P value based on the (3.9) strategy.

By varying the P optimization value between 0 and 100 MW, the results in Fig. 20 were obtained.

When analyzing the imbalance and total profit graphs together, it is evident that both variables show a significant correlation at a 42.8 MW capacity. As seen in Fig. 20, the imbalance value reaches its lowest point at this capacity, indicating the system’s effective control over energy imbalances. This value is approximately 72,556 USD. Simultaneously, the total profit at a 42.8 MW capacity is recorded as 13,495 USD. These two results reveal that a 42.8 MW capacity serves as a critical balance point for optimizing energy management strategies.

#### Profit analysis

**Fig. 21:**
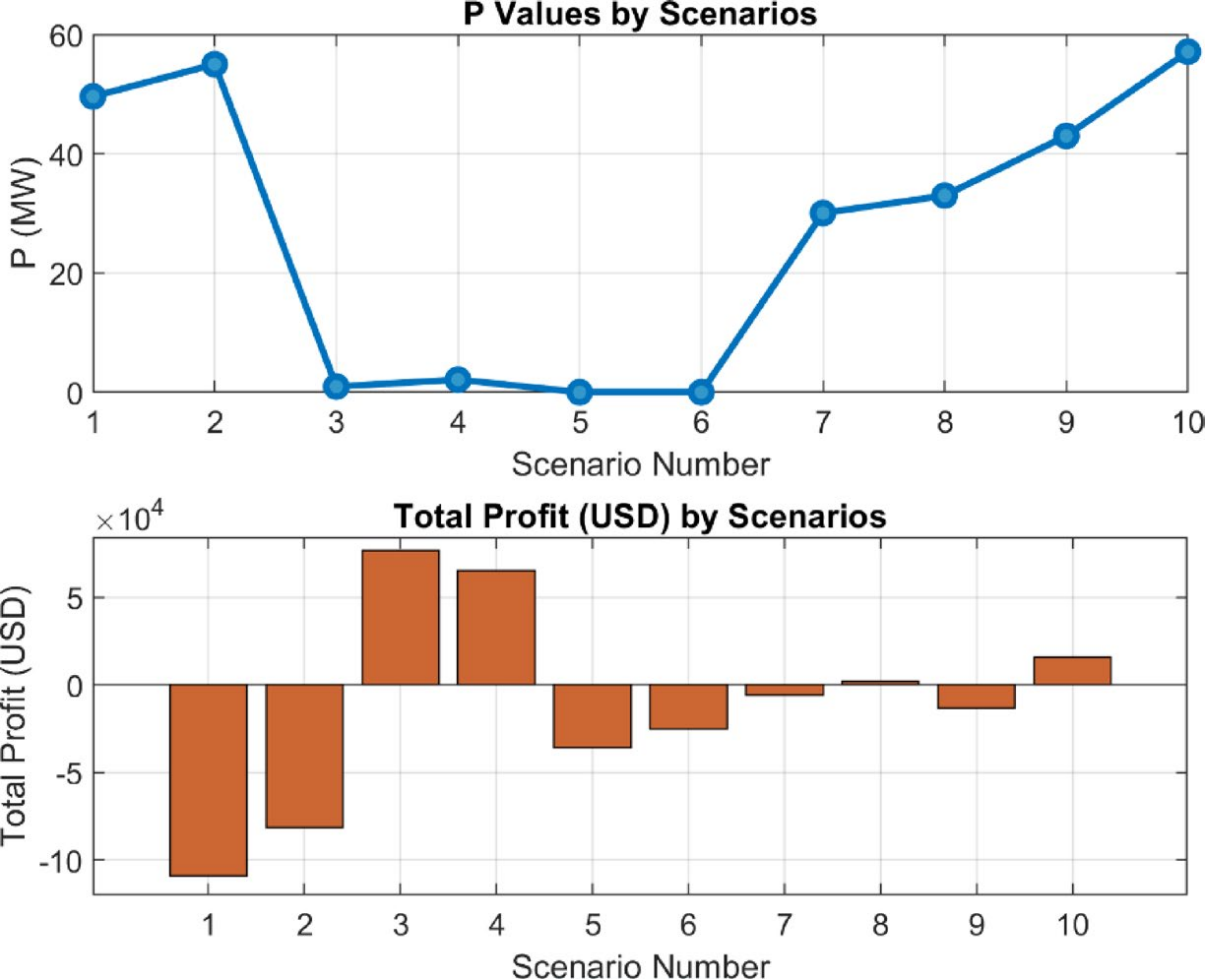
Gain status according to strategies.

In terms of total profit, significant differences between strategies can be observed. The “Operational Strategy Based on Market Electricity Price” and its SOC version achieved the highest total profit values. This indicates that while strategies prioritizing market prices may not fully minimize energy imbalances, they can still generate high profits. Profit focused approaches in strategy selection have been shown to offer higher income potential, especially under fluctuating market conditions. This situation is visually depicted in Fig. 21, where each strategy exhibits distinct profit outcomes.

#### Strategies focused on imbalance and market prices

Some strategies considered both imbalances and market prices. The “Operational Strategy Based on Imbalance and Market Prices” did not minimize the P optimization value but still yielded satisfactory profit results. This strategy demonstrates a balanced approach to managing energy imbalances while leveraging market conditions to generate profit.

#### Performance of SOC versions

SOC versions generally produced distinct profit and imbalance outcomes compared to standard strategies. For instance, the “Market Price Strategy with a Priority on Reducing Imbalance Costs (SOC)” achieved better total profits compared to its standard counterpart, despite having a lower P optimization value. This result underscores the potential of SOC strategies to optimize both imbalances and profits more effectively in energy management.

#### Balance between profit and imbalance

The results show that each strategy generates varying levels of profit and imbalance. High profit strategies often encounter greater energy fluctuations, whereas strategies with lower imbalances produce more modest profit outcomes. This highlights the importance of balancing profit and imbalance control when selecting a strategy.

## Validation of results

To ensure the reliability and applicability of the proposed strategies, a comprehensive validation process was conducted. This section presents an integrated evaluation based on graphical outputs, real world data comparison, sensitivity analysis, and literature benchmarking.

### Graph-based performance validation

The first stage of validation involves the graphical interpretation of simulation results:

- Figure 17(a) shows a significant variation in P (MW) values across strategies, directly influencing downstream economic performance.
- Figure 17(b) and (d) demonstrate that battery integration substantially reduces imbalance costs, especially in Strategies 2 and 3 while increasing total revenue.
- Figure 17(c) and (g) highlight profit components, where Strategies 3 and 4 show the highest profitability, whereas Strategy 2 yields negative total profit, likely due to investment inefficiency.
- **MinCA analysis** in Fig. 17(e) confirms that despite initial capital costs, some strategies still achieve positive net outcomes.
- **Combined performance** in Fig. 17(h) shows Strategies 3, 4, and 10 delivering the most balanced technical and financial benefits.

These results confirm the internal consistency and efficiency of the battery-based management framework.

### Real-world data comparison

To assess the real-world applicability of the strategies, actual production and market data from a private wind farm operating in Karacabey (Turkey) during December 2023 were used:

- Strategies 3 and 4 achieved the highest revenue increase.
- Strategy 3.9 reduced imbalance costs by approximately 40%.
- A battery capacity of 42.8 MW was found to be optimal in terms of both profit and imbalance cost reduction.

These findings demonstrate that simulation-based strategies align well with practical operational behavior.

### Sensitivity analysis and capacity optimization

A sensitivity analysis was performed by varying key parameters such as market price, battery investment costs, and maintenance expenses. Results revealed:

- Even when the Market price fluctuated between 50 and 120 USD/MWh, battery supported strategies consistently outperformed traditional operations.
- An increase in imbalance penalty coefficients further emphasized the economic advantage of using battery systems.
- Strategies incorporating SOC (State of Charge) limits exhibited longer battery life and higher return on investment.

### Literature consistency check

A comparison with recent literature (e.g., ^17,32,34,38^) shows that this study presents a more comprehensive and data driven strategy structure, especially with the integration of simultaneous Market pricing and imbalance penalty modeling. Strategy 5, in particular, outperforms single aspect approaches proposed in prior studies.

## Conclusions

As demonstrated through case studies and graphical analyses, the proposed strategies and precise capacity calculations contribute to enhancing the performance of battery supported energy systems under various conditions. By implementing these strategies, system operations can be optimized, ensuring efficiency across different scenarios. This study underscores the strategic management of battery systems, emphasizing the importance of balancing investment costs with the associated benefits and revenues. Well designed strategies not only enhance profitability amid market price volatility but also play a crucial role in mitigating energy imbalances. Accurate capacity planning prevents unnecessary investments, ensuring the efficient allocation of resources.

While battery storage systems are already widely adopted, the strategies introduced in this study are instrumental in improving their operational efficiency and cost effectiveness. Additionally, the study provides clear guidance on optimizing battery usage during peak electricity demand periods and managing their operation when demand declines. Overall, the proposed strategies offer sustainable and profitable energy management solutions, delivering significant economic and environmental benefits.

## Recommendations for future research

This study highlights the significant potential of battery based strategies in the energy sector while identifying key areas for future exploration. As energy storage technologies continue to evolve, comprehensive investigations into their implications for system capacity, cost efficiency, degradation patterns, and operational performance are becoming increasingly necessary. In particular, comparative analyses involving various battery technologies such as lithium-ion, solid-state, and flow batteries should be expanded to assess their respective impacts on both short-term operational metrics and long-term strategic outcomes under different market scenarios.

Moreover, the dynamic nature of electricity markets necessitates the development of more flexible, data-driven, and adaptive strategies. Future research should focus on integrating artificial intelligence (AI), machine learning (ML), and predictive analytics into energy storage management systems. These advanced techniques can enhance the forecasting of price volatility, grid demand fluctuations, and renewable generation patterns, thereby enabling real time decision-making and automated optimization of charge-discharge cycles. Hybrid models that combine rule-based logic with data driven optimization may offer a promising direction for improving resilience and responsiveness under uncertainty.

Additionally, as environmental sustainability becomes a global priority, the ecological footprint of battery-based strategies must be carefully evaluated. Life cycle assessments (LCAs), material sourcing implications, recycling potential, and end of life management should be incorporated into future analyses. Furthermore, research should investigate the role of battery energy storage systems (BESS) in supporting carbon neutrality goals by quantifying their contribution to emissions reduction and grid decarbonization. By aligning economic performance with environmental responsibility, future strategies can drive the energy transition in a more holistic and sustainable manner.

Finally, interdisciplinary studies combining engineering, economics, policy, and environmental science will be crucial in shaping integrated frameworks for decision-makers. Such studies can help identify trade offs, synergies, and co benefits across technical, financial, and regulatory dimensions, thus enabling the deployment of battery based energy strategies that are not only profitable and reliable but also aligned with long term societal goals.

## Data Availability

The datasets used and/or analyzed during the current study are available from the corresponding author on reasonable request.

## Abbreviations

BESS: Battery Energy Storage System
IDSMS: Intelligent Demand Management System
SOC: State of Charge
PV: Photovoltaic System
MinCA: Capital Expenditures
MinCA’: Enhanced Capital Expenditures
WPP: Wind Power Plant
MCP: The market clearing price the system marginal price
SMP: The system marginal price
MW: Mega Watt
MWh: Mega Watt-Hour
kW: Kilo Watt
kWh: Kilo Watt-Hour
GWEC: Global Wind Energy Council
GW: Giga Watt
GWh: Giga Watt-Hour
IRENA: International Renewable Energy Agency
TÜREB: Turkish Wind Energy Association
YEKDEM: Renewable Energy Resources Support Mechanism
DC: Direct Current
AC: Alternating Current
V2X: With multi directional Vehicle to Everything
DoD: Depth of Discharge
PMC: Power Management Control
MPPT: Power Point Tracking
LCA: Life Cycle Assessment
